# Refining AMOEBA Electrostatics for d‑Glucose and 1→4-Linked d‑Glucose Oligosaccharides

**DOI:** 10.1021/acs.jcim.6c02832

**Published:** 2026-10-02

**Authors:** Luke A. Newman, Riley Ophof, Valerie Vaissier Welborn

**Affiliations:** † Department of Chemistry, 1757 Virginia Tech, Blacksburg, Virginia 24061, United States; ‡ Macromolecules Innovation Institute, Virginia Tech, Blacksburg, Virginia 24061, United States

## Abstract

Carbohydrate simulations are highly sensitive to the treatment of hydroxyl electrostatics because hydration, conformational sampling, and reactivity are governed by dense hydrogen-bonding networks. Here, we examine how oxygen quadrupole scaling affects AMOEBA d-glucose models generated with Poltype2 by comparing the standard 0.7 scaling with full 1.0 scaling for α- and β-d-glucose monosaccharides, 1→4-linked oligomers, and concentrated solutions. Increasing the oxygen quadrupole scaling from 0.7 to 1.0 improves monosaccharide hydration free energies, increases d-glucose–water hydrogen-bond counts and lifetimes, and strengthens hydroxyl electric-field projections without substantially altering hydration-shell structure. In 1→4-linked d-glucose oligomers, full scaling also enhances intramolecular hydrogen bonding, restricts conformational sampling, and produces more organized chain electrostatics. Electric-field decompositions indicate that OH2 and OH3 in α1→4 d-glucose oligomers are strongly coupled to intramolecular hydrogen-bond networks, whereas OH6 remains more water-controlled and solvent-accessible. Diffusion simulations show that both models perform reasonably well at concentrations up to 0.5 mol/kg but overestimate the slowdown of d-glucose mobility at higher concentrations, especially with 1.0 scaling. Overall, full oxygen quadrupole scaling improves local hydration, hydroxyl electrostatics, and diffusion behavior in the lower-concentration regime. Further refinement is needed to balance these stronger interactions against transport properties in highly concentrated carbohydrate solutions, which may be particularly relevant to materials applications.

## Graphical abstract

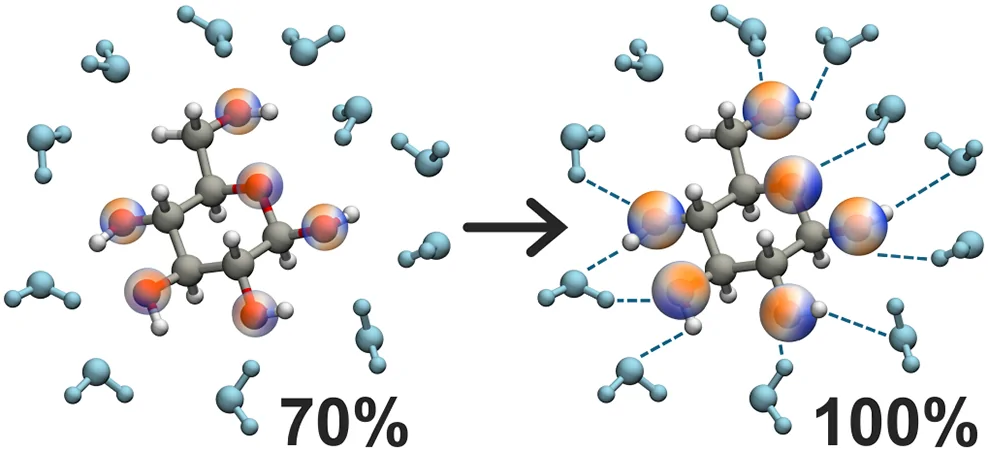

## Introduction

Carbohydrates are a fundamental class of macromolecules that play essential roles in biological systems.^1,2^ They are involved in protein and lipid post-translational modification^3,4^ and mediate cell recognition,^5^ adhesion,^6^ and signaling processes^7^ thereby contributing to host–pathogen interactions^8,9^ and immune responses.^10,11^ Among carbohydrates, d-glucose is one of the most fundamental and widely studied monosaccharides.^12–16^ Indeed, d-glucose serves as a major metabolic fuel through glycolysis,^17^ and is the repeating building block of cellulose, a major structural component of plant cell walls and the most abundant organic polymer.^18^ d-glucose is also the repeating building block of amylose and amylopectin, the principal components of starch, the primary energy-storage polysaccharide in plants;^19^ and glycogen, the principal d-glucose-storage polysaccharide in animals and humans.^20^ Many other biologically relevant mono- and oligosaccharides either directly contain d-glucose as a structural unit or are closely related to it chemically. In many cases, these molecules differ only in hydroxyl stereochemistry, glycosidic linkage positions, or additional functional group substitutions. As a result, d-glucose serves as a useful prototypical system for understanding broader trends in carbohydrate structure, hydration, and intermolecular interactions across a wider family of related saccharide structures.

Accurate computational models of carbohydrates are needed to describe the molecular interactions that govern their structural, chemical, and biological behavior across a range of environments. Carbohydrate-specific parameters have been under development and refinement within major biomolecular force fields such as CHARMM/Drude^21–23^ and GLYCAM.^24,25^ The development of reliable carbohydrate force fields remains challenging because carbohydrates have flexible conformational landscapes^26,27^ and contain highly polar and directional functional groups that give rise to hydration environments with strong hydrogen-bonding networks.^28^ These features make carbohydrates particularly sensitive to the balance of bonded, van der Waals, and electrostatic terms in a molecular mechanics model.

Polarizable force fields are an appealing framework for carbohydrate simulations because they should better represent the directional lone-pair interactions of the hydroxyl groups and will respond to changes in local environment, thereby improving the description of carbohydrate hydration relative to fixed-charge models.^29–33^ The AMOEBA polarizable force field has been shown to perform well for molecular systems with strong polarity and diverse electrostatic environments.^34–39^ In our previous work, we demonstrated that AMOEBA provides a promising baseline description of monosaccharides in water using minimally refined parameters.^40^ Building from that foundation, it is important to further assess how well these parameters describe carbohydrate hydration and conformational properties, and whether they remain transferable from monosaccharides to larger oligomer systems.

Hydration free energy is a useful property for evaluating force field quality because it is sensitive to the balance of solute–water interactions encoded in the underlying model.^41^ It has therefore been used as a benchmark for assessing simulation results between models and importantly with respect to experimental target values.^42–45^

In the development of polarizable force fields, empirical scaling of electrostatic and polarizability parameters derived from gas-phase calculations has sometimes been required to obtain improved condensed-phase behavior. Specifically, the polarizability scaling factor for ethers was increased from 0.7 to 0.85 to better reproduce experimental dielectric constants in the Drude polarizable force field.^46^ An early AMOEBA study found that scaling the oxygen quadrupole moment to 73% of the gas-phase value improved the description of water dimer structure compared to ab initio and experimental results.^47^ Later work from Shi et al. showed that scaling oxygen quadrupole components to 70% of gas phase values resulted in improved accuracy of hydration free energies for small hydroxyl-containing organic molecules.^48^ Similarly, a quadrupole scaling factor of 0.6 was used for ammonia to reproduce the experimental molecular quadrupole moment, and both of these quadrupole scalings were kept consistent for -NH and -OH functional groups within AMOEBA.^49^ Bradshaw and Essex observed an overestimation of hydration free energies for aliphatic alcohol groups in AMOEBA and used a quadrupole scaling factor of 0.6 to obtain a decrease in error to experimental values.^50^

These findings suggest that direct transfer of gas-phase electrostatics into condensed-phase simulations may not always provide the most accurate description of hydration, especially for strong hydrogen-bonding functional groups. Although no broadly standardized scaling protocol has been established for carbohydrate-specific AMOEBA parameterization, the AMOEBA/Poltype2 ^51^ workflow recommends scaling oxygen quadrupole components to 70% of their gas-phase values for non-aromatic hydroxyl and ether oxygens, among other functional groups. In this work we revisit this assumption for d-glucose-based systems by testing different scaling factors for the oxygen quadrupole moments within AMOEBA, and assessing the effects on hydration-related properties. d-glucose is ideal for examining this issue because it differs substantially from the smaller alcohols that motivated earlier quadrupole-scaling studies and contains multiple hydroxyl groups. Here, we use AMOEBA to investigate the hydration behavior of d-glucose monosaccharides, d-glucose solutions over a concentration range of 0.1–4.0 mol kg^–1^, and 1→4-linked d-glucose oligomers up to 10 units in length. We examine the effect of oxygen quadrupole scaling on hydration free energies, diffusion, hydrogen bonding, and local electric field properties. Through this analysis, we assess both the sensitivity of d-glucose hydration to quadrupole scaling in AMOEBA and the robustness of the resulting parameters for modeling longer 1→4-linked d-glucose polysaccharides.

## Methods

### Parameterization

Poltype2 ^51^ was used to generate AMOEBA parameter sets for α- and β-d-glucose monosaccharides. Dihedral scanning and refinement of non-aromatic rings was explicitly enabled, and the newer grid-based Gaussian Distributed Multipole Analysis (GDMA) algorithm was utilized.^52^ Parameters for the 1→4 anomeric linkage were obtained in a modular manner from a disaccharide model system. The parameters involving the glycosidic oxygen, the new bonded and non-bonded terms introduced by the linkage, were extracted and made compatible with the monosaccharide parameter sets. The corresponding Poltype2 input files and all the parameters used in this work are available on the WelbornGroupGitHubpage.

To probe the effect of scaling atomic quadrupoles in AMOEBA, the oxygen quadrupole moments obtained from the gas-phase GDMA results were scaled to generate modified parameter sets. The AMOEBA/Poltype2 default value of 70% gas-phase oxygen quadrupoles (0.7 scaling) was used as a baseline, along with a full oxygen quadrupole scaling parameter variation (1.0 scaling), for the monosaccharide, monosaccharide concentration, and oligomer systems. Additionally, for the single molecule monosaccharide systems two more scaling variations were tested where the hydroxyl oxygens were set to 60% and 100% gas phase values (0.6_OH_ and 1.0_OH_ scaling respectively) while the O5 ether oxygen was held at the default 0.7 scaling.

### System Preparation

Initial saccharide structures were obtained from the GLYCAM-Web polysaccharide builder library^53^ and solvated in cubic water boxes using the Tinker8 software package.^54^ Single α-d-glucose and β-d-glucose monosaccharides were placed in cubic water boxes with a side length of 30 Å. Mixed monosaccharide systems were prepared at concentrations of 0.1, 0.25, 0.5, 1, 2, 3, and 4 mol kg^–1^ (12, 29, 56, 105, 188, 255, and 305 d-glucose) were prepared in 60 Å^3^ water boxes. These systems were constructed using the experimental anomeric ratio of 36% α-d-glucose and 64% β-d-glucose. oligomer systems containing 2, 4, 6, 8, and 10 d-glucose units were also prepared. The cubic box lengths were varied according to system size, using box lengths of 31, 35, 39, 43, and 50 Å for the α series and 31, 43, 45, 63, and 65 Å for the β series. The coordinates of all starting geometries are provided on the WelbornGroupGitHubpage.

### Molecular Dynamics Simulations

All systems were energy minimized and simulated using GPU-enabled Tinker9.^54,55^ Production simulations were performed in the isothermal-isobaric ensemble (NPT) at 300 K and 1 atm using the Nosé–Hoover thermostat and barostat and velocity Verlet integrator. Trajectory frames were saved every 10 ps. Single-monosaccharide systems were simulated for 750 ns, with the first 50 ns discarded as equilibration. Mixed-concentration monosaccharide systems were simulated for 1250 ns, with the first 50 ns discarded as equilibration. oligomer systems were simulated for 600 ns, with the first 100 ns discarded as equilibration. Root-mean-square-deviation and torsional potential energy of each oligomer system is shown in Figures S1–S4. Scripts for diffusion, dihedral angles, and hydrogen bonding utilized the MDAnalysis open-source Python library.^56^

### Diffusion

For the mixed-concentration monosaccharide systems, diffusion calculations were carried out in the canonical ensemble (NVT) as barostat interference has been shown to affect molecular diffusion.^57^ For each concentration, 10 replicate simulations were initiated from the NPT production trajectories at 125 ns intervals. Coordinates and velocities were carried forward from the parent NPT trajectory, and each replicate was simulated for 25 ns in the NVT ensemble at 300 K with the Nosé–Hoover thermostat and velocity Verlet integrator. The average box size was obtained from the NPT production for each concentration and held constant in these replicates. The first 5 ns of each replicate were discarded as equilibration. The mean-square displacement of the d-glucose center of mass was calculated as a function of time, averaged over all d-glucose molecules, and fit using a linear regression (Figure S5). The diffusion coefficient was calculated from the slope of the mean-square displacement using the Einstein (eq 1):

1 $$D=\underset{t\to \infty }{\lim}\frac{\left\langle {|r(t)-r(0)|}^{2}\right\rangle }{6t}$$

where *D* is the diffusion coefficient, ⟨|**r**(*t*) – **r**(0)|^2^⟩ is the mean-square displacement of the d-glucose center of mass averaged over all d-glucose molecules in the simulation, and *t* is time.

A finite-size correction was then applied to the diffusion coefficients according to the equation from Yeh and Hummer (eq 2):^58^

$$D_{corr}=D+\frac{k_{B}T\xi }{6\pi \eta L}$$ 2

where *D*_*corr*_ is the finite-size corrected diffusion coefficient, *D* is the diffusion coefficient obtained directly from the simulation, *k*_B_ is the Boltzmann constant, *T* is the temperature, *ξ* is the Yeh–Hummer constant for a cubic box (*ξ* = 2.837297), *L* is the box length, and *η* is the shear viscosity of the solution at the corresponding d-glucose concentration, obtained from experimental values and extrapolated to the specific concentrations studied.^59^ The finite-size corrections for concentrations of 0.10, 0.25, 0.50, 1.00, 2.00, 3.00, and 4.00 mol kg^–1^ were 1.25, 1.17, 1.04, 0.82, 0.51, 0.32, and 0.22 × 10^–10^ m^2^ s^–1^, respectively.

### Hydration Free Energy Calculations

Hydration free energies (Δ*G*_*hyd*_) for the mono- and oligomers were computed using the thermodynamic cycle shown in Figure 1a. In this approach, the d-glucose molecule was progressively decoupled in both vacuum and aqueous environments through a series of thermodynamic steps.

**Figure 1:**
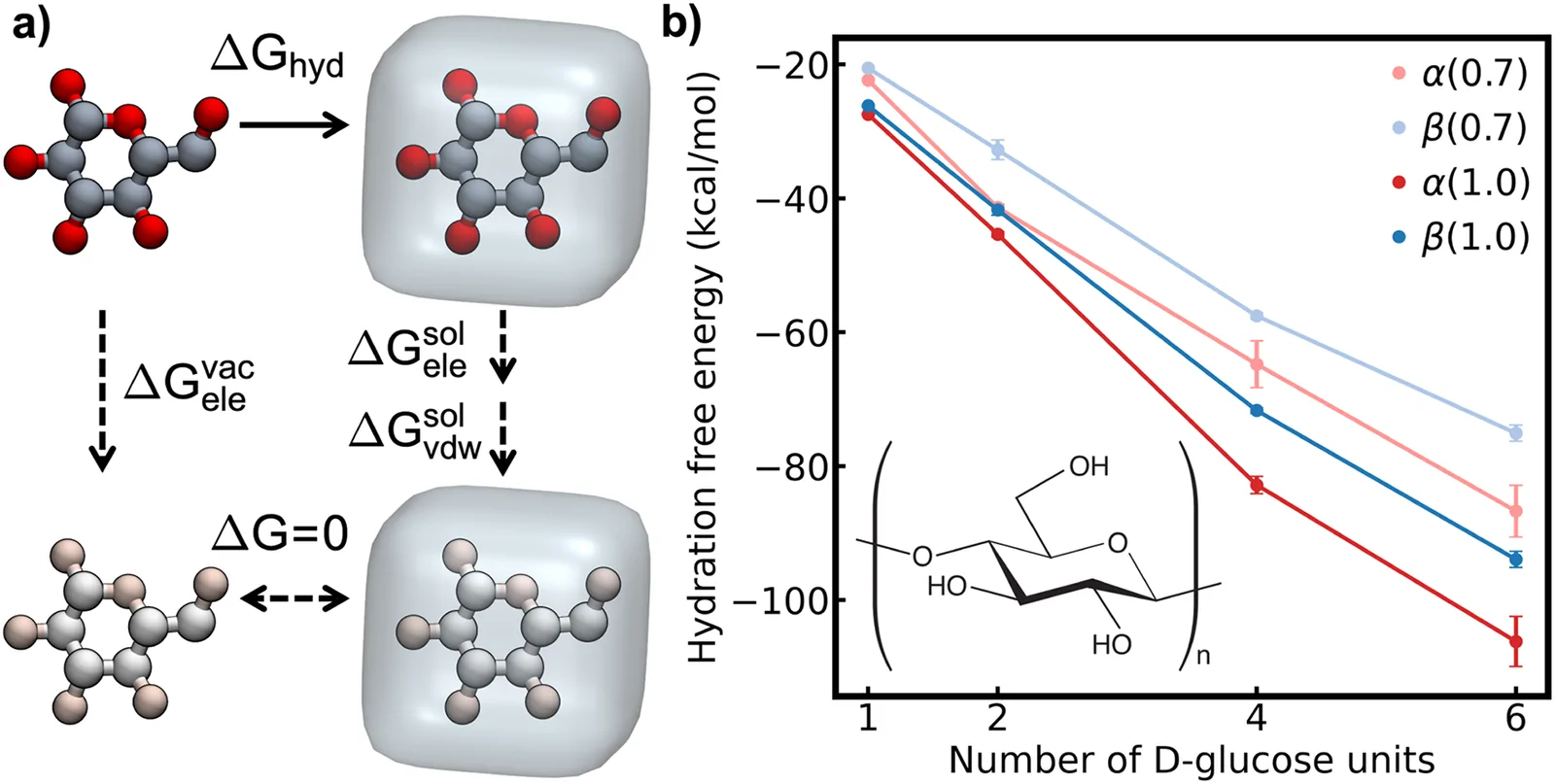
(a) Alchemical free energy cycle used to obtain monosaccharide and oligomer hydration free energies. b) Hydration free energy for α- and β-d-glucose oligomers with increasing chain length. The oxygen quadrupole scaling factor is noted in parenthesis in each case.

In vacuum, the electrostatic interactions of the d-glucose molecule were annihilated over 11 thermodynamic states using the following λ_ele_ schedule:

$$\lambda_{\mathrm{ele}}=\left\{1.0,0.9,0.8,0.7,0.6,0.5,0.4,0.3,0.2,0.1,0.0\right\}$$

Both intra- and intermolecular electrostatic interactions were scaled during this process. Each λ state was simulated independently from the same starting geometry. Vacuum simulations were run in the canonical ensemble (NVT) at 300 K using the velocity Verlet integrator and the Nosé–Hoover thermostat for 3.5 ns, with frames saved every 0.1 ps. For each thermodynamic state, an initial equilibration period of 5000 frames was used, corresponding to 0.5 ns, and a production window of 3 ns.

In water, the free energy cycle consisted of two stages. First, the electrostatic interactions of d-glucose were annihilated over 11 states using the same λ_ele_ schedule as in vacuum while keeping λ_vdW_ = 1.0:

$$\lambda_{\mathrm{ele}}=\left\{1.0,0.9,0.8,0.7,0.6,0.5,0.4,0.3,0.2,0.1,0.0\right\}$$

Second, with λ_ele_ = 0.0, the intermolecular van der Waals interactions between d-glucose and water were decoupled over 18 additional states using the following λ_vdW_ schedule:

$$\begin{array}{ll} \lambda_{\mathrm{vdW}}= & \{0.975,0.95,0.90,0.85,0.80,0.75,0.70,0.65,0.60,\\ & 0.55,0.50,0.40,0.30,0.20,0.10,0.05,0.025,0.0\} \end{array}$$

All aqueous free energy simulations were run in the NPT ensemble at 300 K and 1 atm using the Nosé–Hoover thermostat and barostat for 11 ns, with frames saved every 0.2 ps. For each thermodynamic state, an initial equilibration period of 5000 frames was used, corresponding to 1.0 ns, and a production window of 10 ns.

Free energy differences between neighboring thermodynamic states were calculated using the Bennett acceptance ratio (BAR) method^60^ as implemented in Tinker9. BAR estimates the free energy difference between adjacent states from their respective Boltzmann-weighted energy distributions. Statistical uncertainties can also be obtained from BAR through bootstrapping; however, these uncertainties were found to be small for all hydration free energy calculations in this study, typically less than 0.07 kcal/mol for monosaccharides and 0.2 kcal/mol for oligomers. Accordingly, the uncertainties reported here are based on the variation between replicate calculations, which was larger than the statistical uncertainty. Replicate uncertainties were calculated as the standard deviation across triplicate gas-phase and aqueous-phase calculations, and standard error propagation was then used to combine the gas-phase and aqueous-phase uncertainties.

The hydration free energy (eq 3) was then defined as

3 $$\Delta G_{\mathrm{hyd}}=\Delta G_{\mathrm{ele}}^{\mathrm{vac}}-\left[\Delta G_{\mathrm{ele}}^{\mathrm{sol}}+\Delta G_{\mathrm{vdw}}^{\mathrm{sol}}\right]$$

where $\Delta G_{\mathrm{ele}}^{\mathrm{vac}}$ is the free energy associated with annihilating the electrostatic interactions of d-glucose in vacuum and similarly $\Delta G_{\mathrm{ele}}^{\mathrm{sol}}$ in water, and $\Delta G_{\mathrm{vdw}}^{\mathrm{sol}}$ is the free energy associated with decoupling the d-glucose–water van der Waals interactions.

Replicate hydration free energy calculations were initiated from frames extracted from the longer equilibrium trajectories, carrying forward both coordinates and velocities into the free energy protocol. For the monosaccharides, replicate calculations were initiated from frames at 150, 300, and 600 ns. For the oligomers, replicate calculations were initiated from frames at 200, 400, and 600 ns. For each system, the final hydration free energy (Δ*G*_hyd_) was obtained by averaging the three vacuum contributions, $\Delta G_{\mathrm{ele}}^{\mathrm{vac}}$ , and the three water contributions, $\left[\Delta G_{\mathrm{ele}}^{\mathrm{sol}}+\Delta G_{\mathrm{vdw}}^{\mathrm{sol}}\right]$ , separately before taking their difference according to eq 3. The corresponding uncertainty was obtained by taking the standard deviation of the vacuum contributions and the standard deviation of the aqueous contributions separately and combining these uncertainties through standard propagation of error.

### Electric Field Calculations

Electric field projections along d-glucose hydroxyl bonds were computed using ELECTRIC.^61^ For a hydroxyl bond between oxygen atom O and hydrogen atom H, the projected electric field along the bond was defined as in eq 4:

4 $$E_{\mathrm{proj}}^{\mathrm{OH}}=\frac{E^{O}+E^{H}}{2}\cdot \mu^{\mathrm{OH}}$$

where **E** ^O^ and **E** ^H^ are the electric field vectors at the oxygen and hydrogen atoms, respectively, and **μ** ^OH^ is the unit vector along the O–H bond axis. This analysis was applied to the hydroxyl bonds present in the d-glucose molecules, specifically O_1_–H, O_2_–H, O_3_–H, O_4_–H, and O_6_–H.

### Hydrogen Bonding Analysis

Hydrogen bonds were identified using a geometric criterion consisting of a maximum donor–acceptor distance of 3.5 Å and a minimum donor–H–acceptor angle of 145°. Hydrogen bond counts were calculated over the NPT production simulations for the monosaccharide and oligomer systems.

Hydrogen bond lifetime calculations required a higher frame printout frequency and were therefore carried out using the first thermodynamic state from each of the three hydration free energy replicates. For these simulations, frames were saved every 0.2 ps. Hydrogen bond lifetime autocorrelation functions, *C*(*t*), were computed using the hydrogen bond lifetime analysis implemented in MDAnalysis. A maximum lag time of 100 frames, corresponding to 20 ps, was used to capture sufficient decay of the hydrogen bond populations (Figures S6–S7). An intermittency of 1 frame was applied, such that a hydrogen bond was only considered broken if it remained outside the geometric criterion for 2 or more consecutive frames.

Separate lifetime autocorrelation functions were computed for glucose acting as a hydrogen bond donor to water and for glucose acting as a hydrogen bond acceptor from water. The resulting autocorrelation functions were fit to a stretched exponential (eq 5):

5 $$C\left(t\right)=A\exp\left[-{\left(\frac{t}{\tau }\right)}^{\beta }\right]$$

where *A* is the fitted amplitude, *τ* is the characteristic hydrogen bond lifetime, and β is the stretching exponent.

Fits were performed using nonlinear least-squares optimization with the curve_fit routine from scipy.optimize, using bounded parameter constraints. The fitted parameters were constrained such that *τ* > 0, 0 < β ≤ 1, and 0 ≤ *A* ≤ 1.5. Fits began at *t* = 0 and extended up to, but did not include, the first point at which *C*(*t*) < 0.05. If an autocorrelation function did not fall below 0.05 within the sampled 20 ps interval, all available points were included. The fitted *τ* values from the three replicates were averaged, and uncertainties are reported as the standard error of the mean across replicates. Fits for the individual replicates are shown in Figures S6–S7, and the corresponding *τ*, β, and *R* ^2^ values are reported in Table S1.

## Results and Discussion

### Hydration Free Energy

We present in Table 1 the hydration free energies for α- and β-d-glucose monosaccharides, computed using the alchemical free energy cycle shown in Figure 1a. Individual vacuum, electrostatic-solvation, and van der Waals-solvation free energy contributions for each replicate are reported in Table S2. We show results with varying oxygen quadrupole scaling: 0.6_OH_, 0.7, 1.0_OH_, and 1.0. The 0.7 and 1.0 models correspond to all d-glucose oxygen quadrupoles set to 70% and 100% of their gas-phase GDMA values, respectively. In the 0.6_OH_ and 1.0_OH_ models, the hydroxyl oxygen quadrupoles are set to 60% and 100% of their gas-phase GDMA values, respectively, while the O5 ring oxygen is held at the default 0.7 scaling. We observe that, from the 0.6 to the full 1.0 scaling model, the hydration free energy changes by −6.5 kcal/mol for α-d-glucose and −7.0 kcal/mol for β-d-glucose. Further, we note that changing the scaling factor of the five hydroxyl oxygen quadrupoles from the default 0.7 to 1.0 shifts the hydration free energy by −5.2 and −5.5 kcal/mol for α- and β-d-glucose, respectively. In contrast, scaling only the O5 ether-type oxygen quadrupole by the same magnitude (1.0_OH_ vs. 1.0 models) changes the hydration free energy by less than 0.1 kcal/mol. Overall, our results show that hydration free energies become increasingly negative, corresponding to stronger water affinity, as the hydroxyl quadrupoles are increased toward their full gas-phase values.

**Table 1:** Hydration Free Energies for α-d-Glucose, β-d-Glucose, and an Equilibrium-Weighted α:β Ratio (36:64) under Different Oxygen Quadrupole Scaling Conditions^a^

Hydration free energy (kcal/mol)	0.6_OH_	0.7	1.0_OH_	1.0
α-d-glucose	–20.9 ± 0.2	–22.3 ± 0.1	–27.5 ± 0.2	–27.4 ± 0.1
β-d-glucose	–19.1 ± 0.1	–20.5 ± 0.1	–26.0 ± 0.1	–26.1 ± 0.1
Equilibrium α:β (36:64)	–19.7 ± 0.1	–21.1 ± 0.1	–26.5 ± 0.1	–26.6 ± 0.1

a The 0.7 and 1.0 models correspond to all d-Glucose oxygen quadrupoles set to 70% and 100% of their gas phase GDMA values, respectively. In the 0.6_OH_ and 1.0_OH_ models, the hydroxyl oxygen quadrupoles are set to 60% and 100% of their gas-phase GDMA values, respectively, while the O5 ring oxygen is held at the default 0.7 scaling.

We extend this analysis to examine the hydration free energies of α- and β-d-glucose oligomers as a function of chain length, focusing on the 0.7 and 1.0 quadrupole scaling conditions (Figure 1b). For both anomers and scaling models, we see that hydration free energy becomes more negative, thus favorable, with oligomer length. The oxygen quadrupole scaling produces a length-dependent shift, with longer oligomers and greater scaling factor (1.0) yielding stronger hydration free energies. Overall, we see that each additional d-glucose unit contributes a relatively constant increment to the hydration free energy. Linear fits (Figure S8a) give slopes of −12.6 and −11.0 kcal mol^–1^ per d-glucose unit for the 0.7-scaled α- and β-d-glucose oligomers, respectively. This compares with the steeper −16.0 and −13.6 kcal mol^–1^ per d-glucose unit slopes for the corresponding 1.0-scaled systems. However, after normalizing by the number of oxygen atoms in each oligomer (Figure S8b), the slopes become approximately 0.18–0.25 kcal mol^–1^ per oxygen atom across the four anomer/scaling combinations. Our results therefore indicate that the hydration free energy per oxygen atom becomes slightly less favorable as the fraction of interior d-glucose units increases. Consequently, we do not expect the hydration free energy to continue decreasing at the same rate as the number of units increases. Instead, we anticipate that the hydration free energy will eventually plateau as these two effects offset one another.

Experimental references for the hydration free energy of d-glucose are difficult to determine because of its high solubility and low vapor pressure. As a result, reported values vary depending on the experimental data and thermodynamic assumptions used to derive the target hydration free energy. For example, an experimental target value of −25.5 kcal/mol was adopted for the Statistical Assessment of Modeling of Proteins and Ligands (SAMPL2) challenge.^44,62,63^ This value has since served as a common benchmark for evaluating molecular simulation models of d-glucose hydration. However, another study used the same experimental vapor-pressure data^62^ as the SAMPL2 target, but derived a hydration free energy of −17.8 kcal/mol, presumably due to differences in the extrapolation procedure.^64^ Others reference an experimental target of −25.0 kcal/mol for β-d-glucose and −40.3 for cellobiose (2 units of β-d-glucose).^65^ It should be noted, however, that experimental hydration free energies for d-glucose generally reflect the equilibrated 36:64 aqueous anomeric mixture, rather than separate contributions from the α- and β-d-glucose monomers.

Despite these inconsistencies in experimental targets, the general consensus is that the hydration free energy of d-glucose should be fairly negative, about −25 kcal/mol. However, multiple computational studies tend to underestimate its value.^44,64,66^ In the SAMPL2 challenge, for example, the average predicted hydration free energy of d-glucose was approximately −20 kcal/mol. As a result, d-glucose was identified as a challenging case, with nearly half of the 47 submissions underestimating the hydration free energy by at least 6 kcal/mol.^44^ In contrast, Sauter and Grafmüller reported a more favorable hydration free energy of −26.5 kcal/mol for α-d-glucose using the GLYCAM/TIP5P model, and a hydration free energy of −44.4 kcal/mol for cellobiose.^65^ Similarly, a recent DFT study by Barrios et al. reported a hydration free energy of −44.2 kcal/mol for cellobiose.^67^ With our AMOEBA full-scaling model, we find an equilibrium-weighted hydration free energies of −26.6 kcal mol^–1^ for d-glucose (Table 1) and −41.7 kcal mol^–1^ for cellobiose, bringing the predictions into closer agreement with experiments. Thus, restoring the full gas-phase oxygen quadrupole magnitudes shifts the AMOEBA hydration free energies in the correct direction.

### Hydrogen Bonding in Monosaccharides

We show in Figure 2 the average number of hydrogen bonds between water and α/β-d-glucose with 0.6_OH_, 0.7, 1.0_OH_, and 1.0 oxygen quadrupole scaling. The error bars represent the standard error of the hydrogen-bond count, estimated from block averages computed over seven 90 ns trajectory segments separated by 10 ns gaps to reduce correlation between samples. The resulting standard errors were on the order of 0.005 hydrogen bonds and were not significantly changed by the block size chosen. In Figure 2, we see a gradual increase in average number of hydrogen bonds with oxygen quadrupole scaling, more so in the average number of acceptor than donor hydrogen bonds. In total (gray numbers in Figure 2), we count more than one additional hydrogen bond for both anomers going from 0.6 to 1.0 scaling. At full scaling, we count a total of approximately 11 hydrogen bonds per d-glucose molecule. This number is consistent with the 11 to 12 hydrogen-bonded waters reported in *ab initio* molecular dynamics studies of d-glucose.^28,68^ Note, however, that the hydrogen bond distance and angle criteria vary between studies, making comparisons approximate.

**Figure 2:**
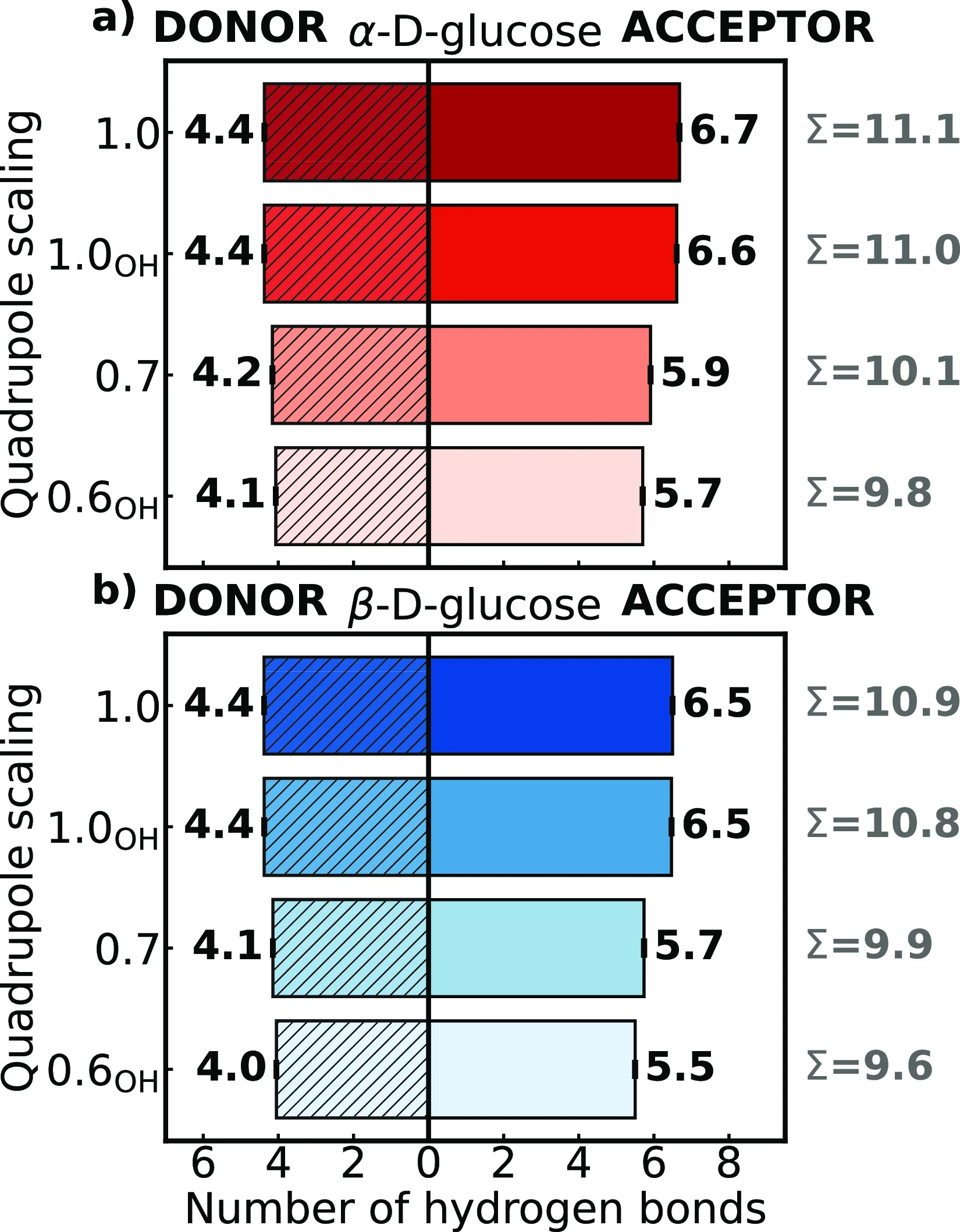
Average donor and acceptor hydrogen bond counts for monosaccharides of a) α-d-glucose (in red) and b) β-d-glucose (in blue) with 0.6_OH_, 0.7, 1.0_OH_, 1.0 quadrupole scaling. Hydrogen bonding to water molecules was counted with a maximum donor-acceptor oxygen distance of 3.5 Å and a donor-hydrogen-acceptor angle greater than 145°. Error bars represent the standard error, calculated from block-averaged hydrogen-bond counts using 90 ns blocks separated by 10 ns gaps.

To better understand how the increase in total hydrogen bonding is distributed across the d-glucose molecule, we compute site-resolved hydrogen-bond counts (Figure S9). For both anomers, increasing the hydroxyl-oxygen quadrupole scaling from 0.6_OH_ to 1.0_OH_ generally increases the number of water-to-d-glucose acceptor hydrogen bonds at the hydroxyl oxygens by 0.12–0.33 hydrogen bonds per site. The ring ether oxygen O5 is an exception: its acceptor hydrogen-bond count decreases over the same hydroxyl-scaling series, by 0.12 hydrogen bonds in α-d-glucose and 0.19 hydrogen bonds in β-d-glucose. When the O5 ether-oxygen multipole is also restored in the full 1.0 model, the O5 acceptor trend flips, increasing by 0.12 and 0.22 hydrogen bonds for the α and β anomers, respectively. This increase is accompanied by compensating decreases at selected hydroxyl oxygen sites, most notably O6 in α-d-glucose (−0.04 hydrogen bonds) and O1 and O6 in β-d-glucose (−0.10 and −0.06 hydrogen bonds, respectively). The donor-side contributions are less site-specific, increasing less strongly with quadrupole scaling across the hydroxyl donor groups, OH1, OH2, OH3, OH4, and OH6, by 0.04–0.09 hydrogen bonds per site.

To further characterize the water–d-glucose hydrogen bonds modeled in AMOEBA, we calculated their lifetimes (Figure 3). The lifetime error bars represent the standard error fitted *τ* values across replicate simulations as described in Methods section. Unlike the hydrogen bond counts, the lifetimes show a more pronounced separation between the lower- and the higher scaling factors. For α-d-glucose, the donor lifetime increases from 4.4–5.0 ps in the 0.6_OH_ and 0.7 models to 8.5 ps in both the 1.0_OH_ and 1.0 models. In contrast, the corresponding acceptor lifetimes increases by less than 1 ps for both anomers.

**Figure 3:**
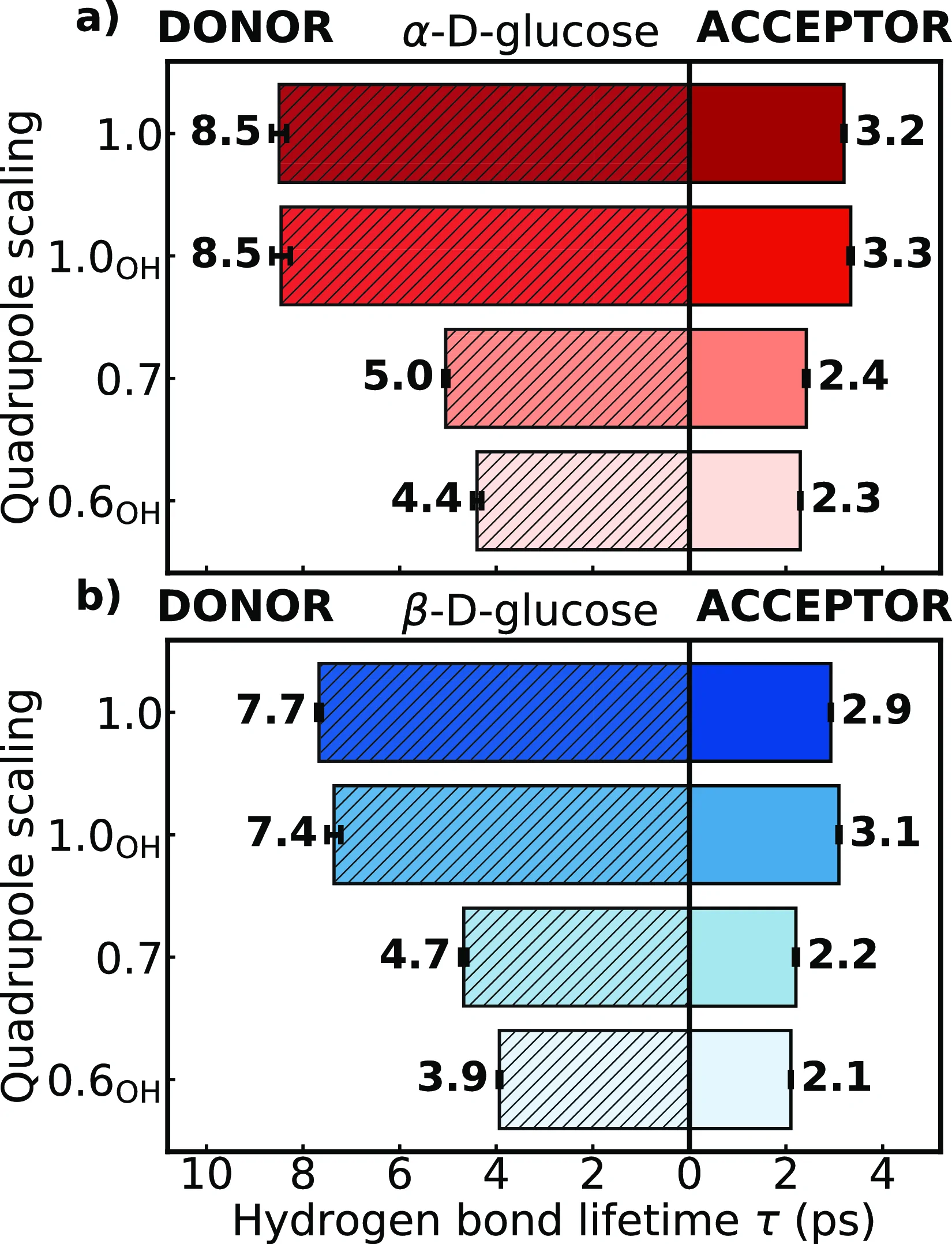
Average donor and acceptor hydrogen bond lifetimes for a) α- and b) β-d-glucose monosaccharides across 0.6_OH_, 0.7, 1.0_OH_, 1.0 quadrupole scaling. Error bars represent the standard error fitted lifetime, *τ*, across three replicate simulations (Methods).

Consequently, we show that increasing the oxygen quadrupole magnitude increases the number of water–d-glucose acceptor hydrogen bonds, but the lifetime response is strongest for donor interactions. The oxygen quadrupole moment helps describe the anisotropic electron density around the oxygen atom, including the lone-pair region that directly participates in acceptor hydrogen bonding. Therefore, scaling the oxygen quadrupole is expected to most directly affect hydrogen bonds in which the d-glucose oxygen acts as the acceptor, consistent with the increase in acceptor hydrogen-bond counts. Counterintuitively, however, the lifetime data indicate that the larger dynamical effect occurs not for acceptor hydrogen bonds, but for hydrogen bonds donated by the hydroxyl group to water. This suggests that the oxygen quadrupole modifies the electrostatic environment of the entire hydroxyl group, increasing hydrogen-bond counts at the acceptor oxygen while also stabilizing hydrogen bonds donated from the hydroxyl hydrogen. Note, however, that the hydration structure around d-glucose is largely preserved across oxygen quadrupole scalings (Figures S10–S14). This interpretation is also consistent with the fitted β values (Table S1), which are approximately 0.75 for acceptor hydrogen bonds and 0.85 for donor hydrogen bonds. The smaller β values for acceptor hydrogen bonds may reflect greater heterogeneity in the geometries available when water donates a hydrogen bond to a d-glucose oxygen. In contrast, hydrogen bonds donated by a d-glucose hydroxyl are constrained by the orientation of a specific O–H bond, resulting in a somewhat narrower distribution of relaxation times. Taken together, these results suggest that quadrupole scaling primarily increases the range of favorable configurations for hydrogen-bond formation from water to d-glucose, while strengthening and prolonging hydrogen bonds donated from d-glucose to water.

To place these lifetime trends in context, we compared the 1.0-scaled AMOEBA results with previously reported carbohydrate–water hydrogen-bond dynamics. Báko et al. used *ab initio* molecular dynamics to report donor/acceptor lifetimes of 5.1/2.0 ps for α-d-glucose and 8.2/3.3 ps for β-d-glucose.^28^ The 1.0-scaled AMOEBA model agrees well with these values for β-d-glucose, but shows poorer agreement for α-d-glucose. Because the *ab initio* simulations were limited in length, this apparent anomeric discrepancy may reflect under-sampling rather than a systematic difference in the underlying hydrogen-bond dynamics. Additional comparison can be made to the force-field study of Pandey and Mallajosyula, who examined carbohydrate–water hydrogen-bond lifetimes for α- and β-d-glucose using several models, including the polarizable Drude force field with the SWM4 water model.^31^ For d-glucose–water hydrogen bonds, they found that the Drude model gave the longest lifetimes, ranging from 3.8–7.1 ps for α-d-glucose and 4.2–7.2 ps for β-d-glucose. Although Pandey et al. used a slightly tighter donor–H–acceptor angle criterion and did not separate donor and acceptor hydrogen bonds, their reported lifetimes fall between our donor and acceptor lifetimes for the 1.0-scaled AMOEBA model. Thus, the 1.0-scaled AMOEBA lifetimes are broadly consistent with prior polarizable and *ab initio* descriptions of d-glucose hydration dynamics, while also revealing a donor/acceptor asymmetry that is not resolved in all previous analyses.

### Hydrogen Bonding in Oligomers

We next examine oligomers, where both intermolecular hydrogen bonding with water and intramolecular hydrogen bonding between d-glucose units contribute. In Figure 4, we show the average number of hydrogen bonds for α- and β-d-glucose oligomers containing 2–10 d-glucose units with 0.7 and 1.0 oxygen quadrupole scaling. The error bars represent the standard error of the hydrogen-bond count, estimated from block averages computed over five 90 ns trajectory segments separated by 10 ns gaps to reduce correlation between samples.

**Figure 4:**
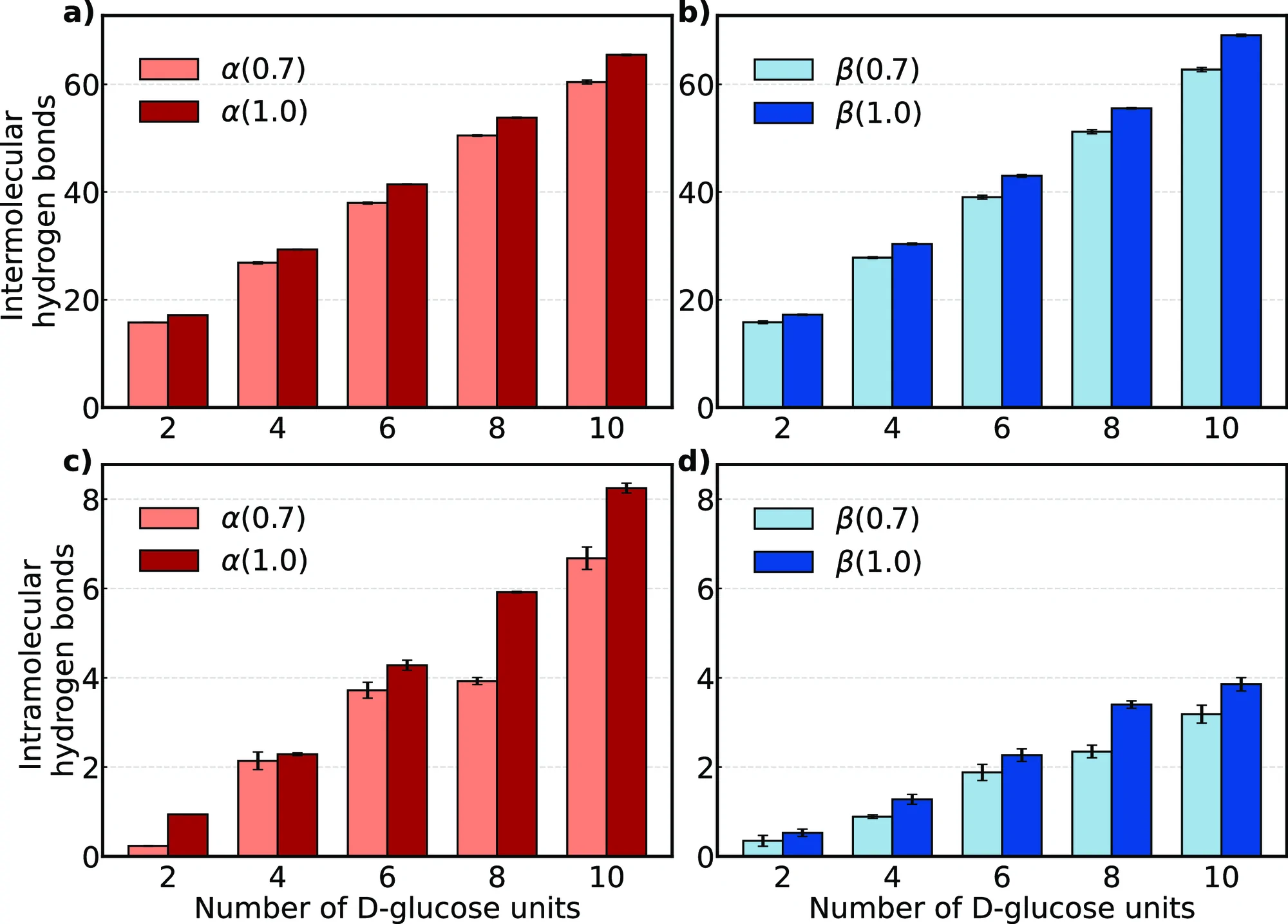
Hydrogen-bond counts for α- and β-d-glucose oligomers containing 2–10 d-glucose units. Intermolecular hydrogen bonds between the oligomer and water are shown for a) α-d-glucose and b) β-d-glucose, while intramolecular hydrogen bonds within the oligomer are shown for c) α-d-glucose and d) β-d-glucose. Lighter-colored bars correspond to 0.7 oxygen quadrupole scaling, and darker-colored bars correspond to 1.0 oxygen quadrupole scaling. Error bars represent the standard error, estimated from block averages computed over 90 ns trajectory segments separated by 10 ns gaps.

We observe that oxygen quadrupole scaling increases the number of hydrogen bonds the oligomers form with water for both anomers. For the α-d-glucose oligomers (Figure 4a), this increase rises from 1.4 additional water hydrogen bond for the 2-unit oligomer to 5.1 additional water hydrogen bonds for the 10-unit oligomer. For the β-d-glucose oligomers (Figure 4b), the rise goes from 1.4 for the 2-unit oligomer to approximately 6.4 additional water hydrogen bonds for the 10-unit oligomer. The per-oxygen contributions to intermolecular oligomer–water hydrogen bonding (Figure S15) show that in the α-d-glucose oligomers, OH3 and OH6 make the largest donor contributions while O2 and O6 make the largest acceptor contributions. In β-d-glucose oligomers, OH3 and OH6 are the dominant donor groups, while O3 and O6 are the dominant acceptor sites. Further, our site-resolved analysis shows that the increase in oligomer–water hydrogen bonding with quadrupole scaling is primarily reflected in the acceptor hydrogen-bond counts, consistent with the monosaccharide results.

For intramolecular hydrogen bonding, the effect of quadrupole scaling differs between the two anomers. In α-d-glucose oligomers (Figure 4c), scaling primarily affects longer oligomers: with 8 units, the average number of intramolecular hydrogen bonds increases from 3.9 to 5.9, while it increases from 6.7 to 8.2 with 10 units. The per-oxygen contributions (Figure S16) show that the internal hydrogen-bond network in the α-d-glucose systems is dominated by OH2 acting as a donor and O3 acting as an acceptor. In the 1.0-scaled 10-unit α oligomer, the OH2 donor and O3 acceptor contributions are 7.6 and 7.5 hydrogen bonds, respectively. In contrast, the average number of intramolecular hydrogen bonds for β-d-glucose oligomers (Figure 4d) remains below 4 even for the 10-unit oligomer. The largest internal contributions in the β oligomers arise from OH2 as a donor and O6 as an acceptor (Figure S16), but at minimal levels with average contributions of 2.5 and 2.6 hydrogen bonds, respectively, in the 1.0-scaled 10-unit oligomer. Note that these findings appear to differ from previous simulations that identified OH3–O5 as the dominant internal hydrogen bond in β-d-glucose oligomers.^65^

Overall, our results show that increasing the oxygen quadrupole magnitude strengthens not only hydrogen bonding with water but also internal hydrogen-bond formation within d-glucose oligomers. This more extensive hydrogen-bond network constrains the accessible conformational space sampled during the simulations. For β-d-glucose oligomers, the 1.0-scaled systems sample a narrower range of conformations than the 0.7-scaled systems and remain closer to their starting structures (Figures S17–S18). The β oligomers also retain a relatively extended geometry, which limits opportunities for intramolecular hydrogen-bond formation. As chain length increases, the greater gain in intermolecular than intramolecular hydrogen bonding is accompanied by an increase in radius of gyration (Figure S19). A reduction in conformational flexibility is also observed for the α-d-glucose oligomers, although their radius of gyration remains largely unchanged with increased quadrupole scaling (Figures S20–S21). Increasing the quadrupole scaling from 0.7 to 1.0 gives structures that better preserve a fixed helix radius (Figures S22–S23), indicating a more constrained helical architecture. However, the α oligomers also undergo a notable conformational rearrangement: the initially left-handed helix rapidly transitions to a right-handed helical conformation, which remains favored throughout the trajectory. To further quantify how oxygen quadrupole scaling affects inter-residue flexibility, we analyzed the pooled *ϕ*_H_/*ψ*_H_ angle distributions across oligomer systems containing 2–10 d-glucose units (Figure 5). The corresponding distributions for the individual systems are provided in Figures S24–S27 and the corresponding J-couplings in Table S3.

**Figure 5:**
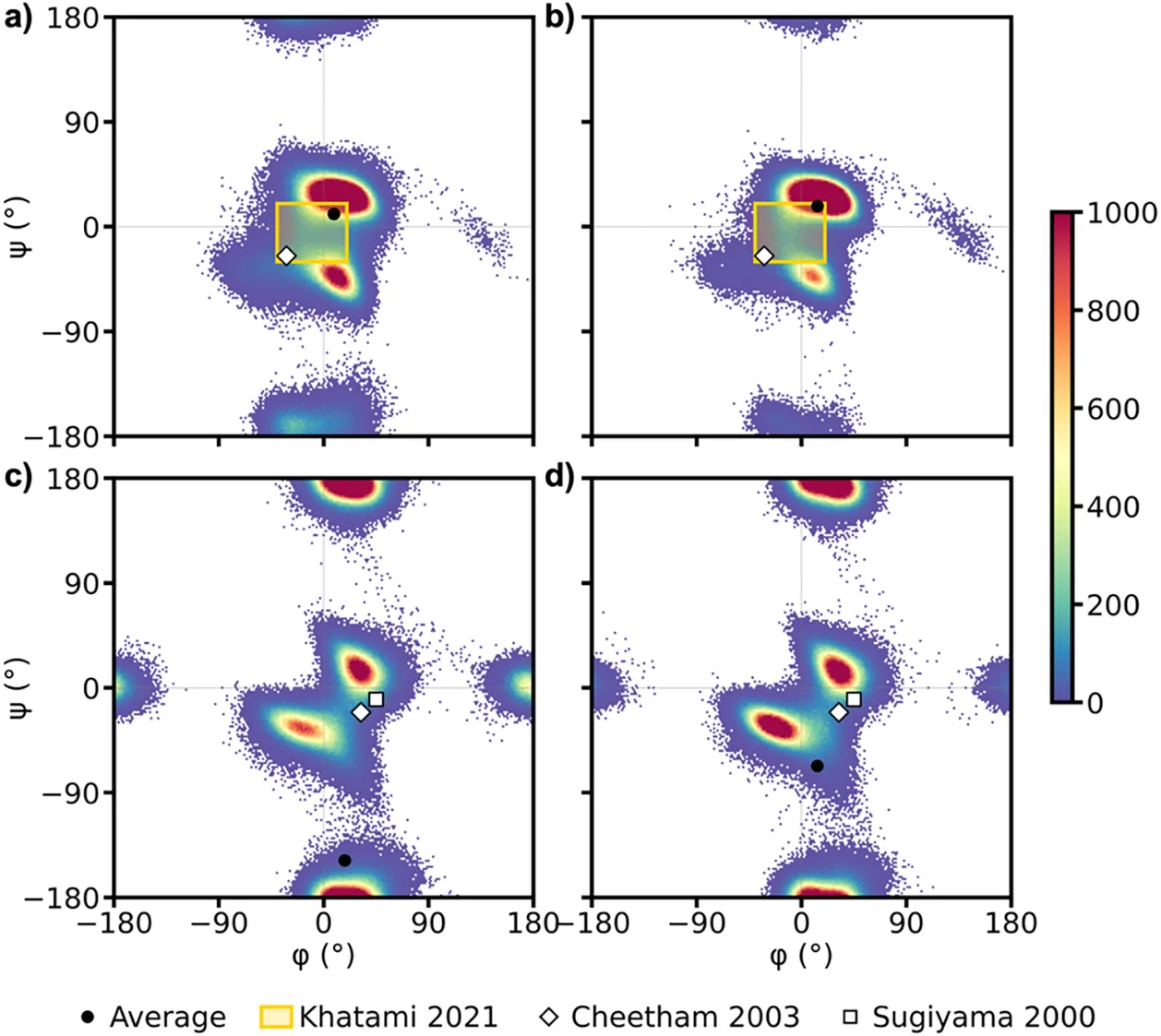
Density plots of the glycosidic dihedral angles *ϕ*_H_ (H1–C1–O–C4) and *ψ*_H_ (C1–O–C4–H4) for α-d-glucose oligomers with a) 0.7 and b) 1.0 oxygen quadrupole scaling, and β-d-glucose oligomers with c) 0.7 and d) 1.0 oxygen quadrupole scaling. The black point denotes the average *ϕ*_H_/*ψ*_H_ angle pair for each pooled distribution. The angles were pooled across all glycosidic linkages in oligomer systems containing 2–10 d-glucose units.

For the α-d-glucose oligomers, both quadrupole-scaling models sample a dominant conformational basin centered near *ϕ*_H_ ≈20° and *ψ*_H_ ≈20° (Figure 5a,b). This basin partially overlaps with the imperfect helical conformational range identified by Khatami et al. in their structural study of amylose.^26^ More broadly, the AMOEBA model samples regions of glycosidic conformational space comparable to those reported for fixed-charge CHARMM and polarizable Drude carbohydrate models.^69^ However, solution NMR measurements by Cheetham et al. suggest an average angle pair closer to *ϕ*_H_ = −32° and *ψ*_H_ = −25°.^70^ The displacement between this experimental estimate and the simulated dominant basin indicates that the linkage torsional parameters may require further refinement, independent of the oxygen quadrupole scaling.

In addition to the dominant basin, lower-density conformations are sampled at more negative *ψ*_H_ values and near the periodic boundary around ± 180°. These alternate conformers are slightly more populated in the 0.7-scaled model, consistent with its broader conformational sampling.

The β-d-glucose oligomers exhibit broader and more heterogeneous conformational distributions than the α systems (Figure 5c,d). Nevertheless, the 1.0-scaled β model gives an average geometry near *ϕ*_H_ ≈ 14° and *ψ*_H_ ≈ −67°, which is closer to experimental values reported by Cheetham et al. and Sugiyama et al.^70,71^

### Consequences for Hydroxyl Reactivity in Solution

Thus far, our results show that increasing the oxygen quadrupole moments in d-glucose systems alters both intra- and intermolecular hydrogen bonding, while largely preserving the structure of the hydration shell (Figures S10–S14). These changes are reflected in more restricted conformational sampling and reduced access to alternate conformational basins. To assess whether this altered hydrogen-bonding and conformational behavior also influences hydroxyl accessibility and potential reactivity, we calculated electric-field projections along each hydroxyl bond. Figure 6 shows these projections for α- and β-d-glucose monosaccharides and 10-unit oligomers in water, with the corresponding results for shorter oligomers provided in Figure S28.

**Figure 6:**
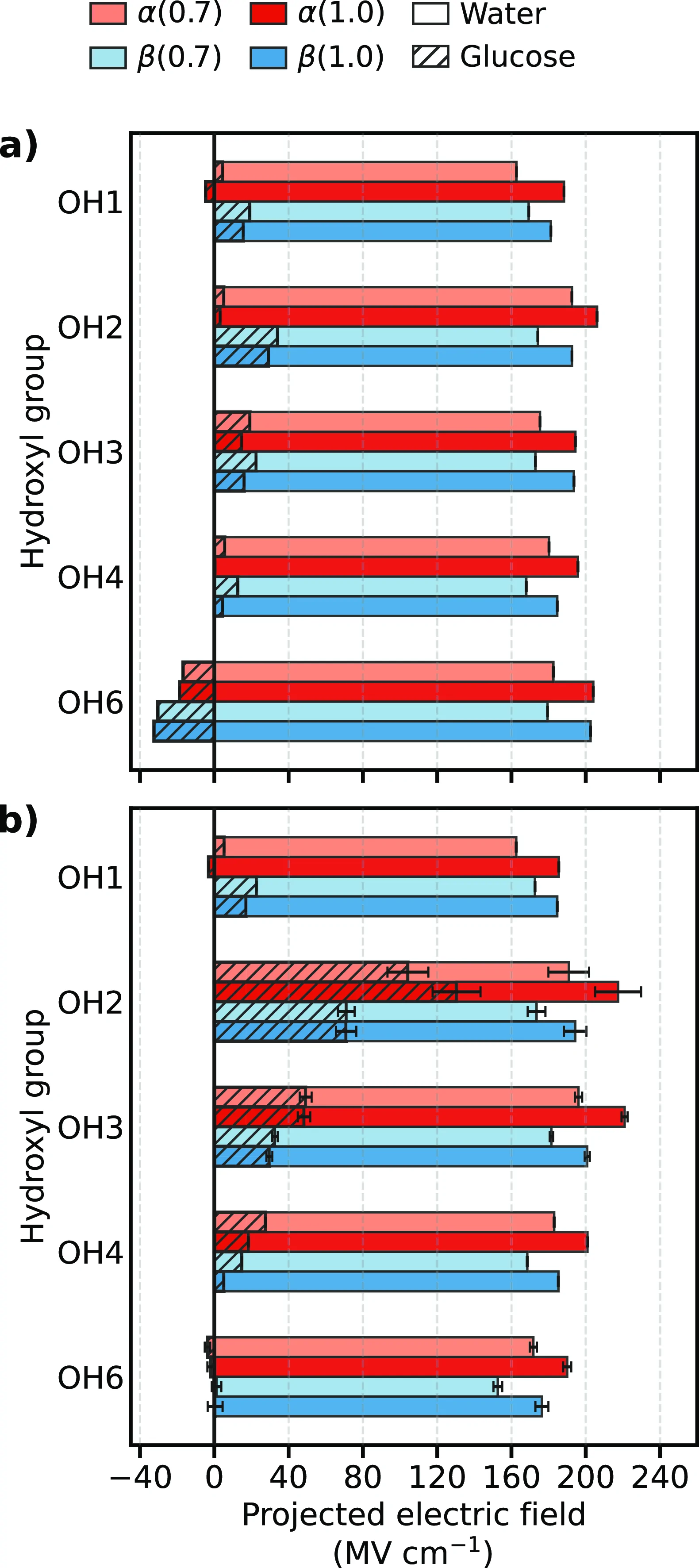
Decomposition of the projected electric field along each hydroxyl bond into water and glucose contributions for α- and β-d-glucose a) monosaccharide and b) 10-unit oligomers. For the monosaccharides, error bars show the standard error over trajectory frames. For the 10-unit oligomers, error bars show the standard error between repeated hydroxyl groups for OH2, OH3, and OH6, and the standard error over trajectory frames for the non-repeated OH1 and OH4 groups.

For both anomers and across chain lengths, increasing the hydroxyl oxygen quadrupole scaling from 0.7 to 1.0 substantially increases the electric-field projections along the hydroxyl bonds, typically by approximately 15–30 MV cm^–1^. At 1.0 scaling, the projected fields exceed 180 MV cm^–1^ at most hydroxyl sites, indicating that d-glucose hydroxyl groups experience highly polarized local electrostatic environments. These field magnitudes are large relative to many electric fields reported in enzymatic active sites, emphasizing the strong electrostatic activation of hydroxyl groups in aqueous carbohydrate environments. The increase in projected field with quadrupole scaling is consistent with the more favorable hydration free energies, larger hydrogen-bond counts, and longer donor hydrogen-bond lifetimes observed for the higher-quadrupole models.

At 1.0 scaling, the isolated monosaccharides show primarily solvent-controlled anomeric differences at OH1, OH2, and OH4, where the water contributions are larger for the α anomer by approximately 23, 40, and 15 MV cm^–1^, respectively. In contrast, the two anomers are much more similar at OH3 and OH6, with water contributions differing by only approximately 1–2 MV cm^–1^.

In the 10-unit oligomers, the largest total projected fields occur in the interior of the α oligomer, particularly at OH3 and OH2. Averaged over units 2–9, the total fields are 224.0 MV cm^–1^ at OH3 and 219.2 MV cm^–1^ at OH2 for the α oligomer, compared with 201.7 and 194.0 MV cm^–1^, respectively, for the β oligomer. However, these large total fields do not arise simply from stronger interactions with water. Instead, oligomerization redistributes the field from a largely solvent-dominated contribution in the monosaccharides to a substantial d-glucose/polymer-derived contribution in the oligomers. This redistribution is most pronounced at OH2 in the α oligomer. At unit 1, the α-OH2 field remains largely water-derived, with a water contribution of 197.1 MV cm^–1^ and a d-glucose contribution of 17.3 MV cm^–1^. In the interior units 2–9, however, the water contribution decreases sharply to 73.0 MV cm^–1^, while the d-glucose contribution increases to 146.2 MV cm^–1^. At unit 10, the redistribution is intermediate, with 88.8 MV cm^–1^ from water and 117.8 MV cm^–1^ from d-glucose. A similar trend is observed in the β oligomer, although the d-glucose/polymer contribution is smaller and the total field enhancement is less pronounced.

OH6 behaves differently from OH2 and OH3. In the α oligomer, the OH6 total field changes only modestly from unit 1 to the interior to unit 10, with values of 186.0, 188.0, and 190.2 MV cm^–1^, respectively. The d-glucose contribution is small or negative across these positions, with values of −4.5 MV cm^–1^ at unit 1, −0.1 MV cm^–1^ in the interior, and −15.1 MV cm^–1^ at unit 10. This indicates that OH6 remains largely water-controlled rather than strongly coupled to the intramolecular chain field. The same general behavior is observed for β-OH6, where the total fields are 174.4, 177.1, and 172.9 MV cm^–1^ for unit 1, units 2–9, and unit 10, respectively. This weaker coupling to the intramolecular field is consistent with experimental observations that often identify OH6 as the most reactive hydroxyl.

Together, these decompositions suggest that large projected fields at OH2 and OH3 reflect an ordered intramolecular electrostatic environment rather than simple chemical accessibility. In the α oligomer, the large d-glucose-derived fields at OH2 and OH3 are consistent with their participation in intramolecular hydrogen bonding, which may reduce their availability for intermolecular reaction. By contrast, OH6 is less strongly involved in intramolecular hydrogen bonding, remains more solvent-exposed, and is therefore more likely to be accessible for intermolecular interactions. Overall, the electric-field analysis suggests that oxygen quadrupole scaling modulates not only oligomer conformation, but also hydroxyl accessibility and potential reactivity, with the clearest effect emerging at OH6.

### Diffusion Kinetics

In this final section, we examine whether oxygen quadrupole scaling also affects the translational diffusion of d-glucose in solution. To this end, we modeled d-glucose solutions over a range of concentrations, from 0.1–4.0 mol kg^–1^. This concentration series was intended primarily as a systematic test of force-field behavior as intermolecular contacts become increasingly frequent, rather than as a direct mapping onto specific physiological glucose concentrations.

Figure 7a shows the finite-size-corrected diffusion coefficients of d-glucose as a function of concentration for AMOEBA simulations using the 0.7 and 1.0 oxygen quadrupole scaling models. The anomer-resolved version is shown in Figure S29. Figure 7b expands the dilute-concentration region and includes additional experimental reference measurements.^72–74^ Data points represent replicate averages, with error bars showing the standard error of the mean from the ten independent NVT replicate simulations. Experimental diffusion data from Gladden and Dole are shown for comparison across this concentration range.^75^ We observe that the simulated d-glucose diffusion coefficients decrease with increasing concentration for both oxygen quadrupole scaling models, consistent with the experimental trend. The full 1.0 scaling model gives systematically lower diffusion coefficients than the 0.7 scaling model at every concentration. In the lower-concentration regime examined here (i.e., ≤0.5 mol kg^–1^), which encompasses the upper range of bulk glucose concentrations relevant to biological systems, both AMOEBA scaling models are close to the experimental reference values. At 0.1 mol kg^–1^, the 0.7-scaled simulations give *D* = 7.10 ± 0.26 × 10^–10^ m^2^ s^–1^, while the 1.0-scaled simulations give *D* = 6.63 ± 0.40 × 10^–10^ m^2^ s^–1^. The experimental references in this dilute range cluster near 6.2–6.7 × 10^–10^ m^2^ s^–1^,^72–74^ placing the 1.0 scaling result closer to the dilute-limit measurements.

**Figure 7:**
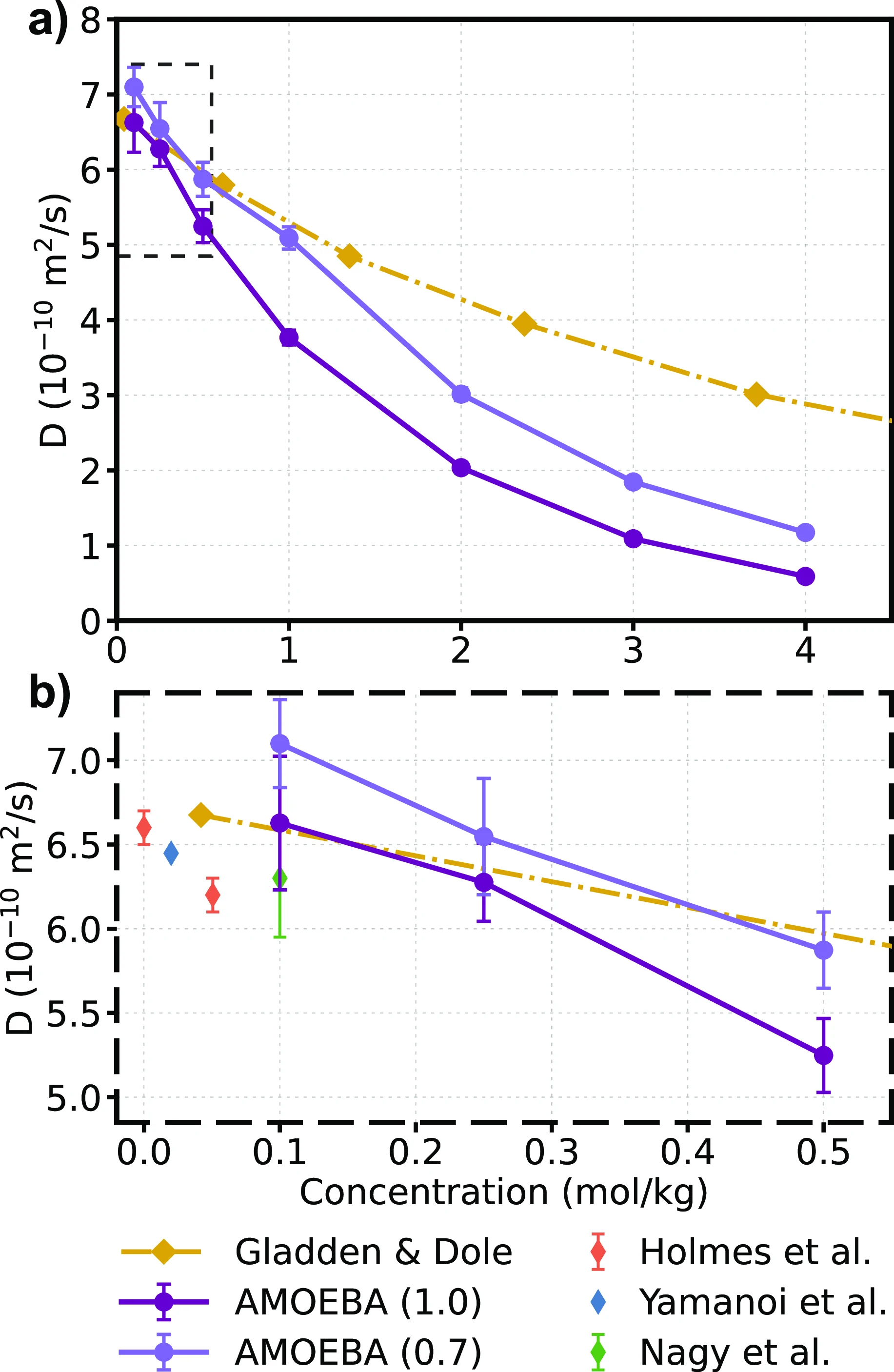
Finite-size-corrected diffusion coefficients of d-glucose as a function of d-glucose concentration. a) Full concentration range from 0.1–4.0 mol kg^–1^ and b) expanded dilute-concentration region from 0.1–0.5 mol kg^–1^ with experimental reference values.

Although the two models show little difference at lower concentrations, their behavior diverges at higher d-glucose concentrations. The molar-to-multimolar portion of this series extends beyond typical bulk physiological d-glucose concentrations and therefore serves primarily as a test of force-field behavior under increasingly crowded solution conditions, while also probing regimes that may be relevant to concentrated carbohydrate formulations and materials applications. Representative snapshots from each simulated concentration are provided in Figure S30 to illustrate the increasing degree of molecular crowding across the concentration series. At 4.0 mol kg^–1^, the 0.7-scaled model predicts a diffusion coefficient of *D* = 1.17 ± 0.02 × 10^–10^ m^2^ s^–1^, whereas the 1.0-scaled model gives a substantially lower value of *D* = 0.59 ± 0.01 × 10^–10^ m^2^ s^–1^. Thus, increasing the oxygen quadrupole scaling leads to slower translational diffusion under concentrated solution conditions, where stronger d-glucose–water and d-glucose–d-glucose interactions are amplified by more frequent intermolecular contacts. Both models underestimate the experimental diffusion coefficient, which is closer to *D* = 3 × 10^–10^ m^2^ s^–1^, indicating that the simulations overestimate the concentration-dependent slowdown in glucose mobility.

One possible explanation for the underestimated diffusion coefficients is that the simulations might sample incorrect d-glucose conformations, which could alter molecular shape and therefore mobility. Previous carbohydrate parameterization studies with the Drude polarizable force field reported oversampling of the higher-energy chair after introducing polarization,^22,76^ and we observed related chair-conformer instabilities in our previous AMOEBA study of monosaccharides.^40^ We therefore examined whether similar conformational artifacts are present here by using the C1–C2–C3–C4 dihedral angle as a diagnostic for the chair conformer population (Figure 8a).

**Figure 8:**
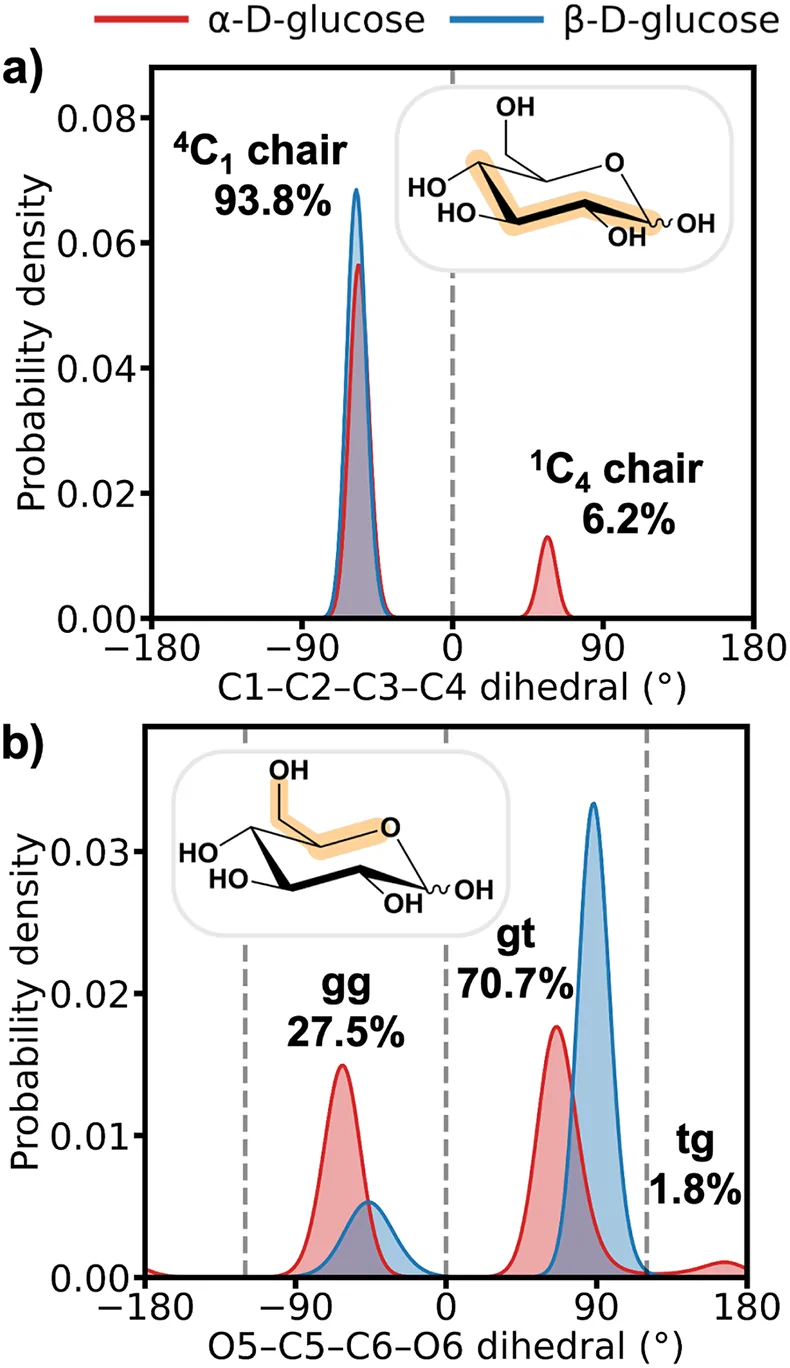
Populations of a) the C1–C2–C3–C4 chair diagnostic dihedral and b) the hydroxymethyl rotamer dihedral, *ω* (O5–C5–C6–O6), for d-glucose monosaccharides. Data were pooled across aqueous d-glucose concentration systems from 0.1–4.0 mol kg^–1^ and include both the 0.7 and 1.0 oxygen quadrupole scaling models.

Across the full dataset, only 6.2% of sampled d-glucose molecules occupy the higher-energy ^1^C_4_ chair conformation, while the ^4^C_1_ chair remains strongly dominant. This population is anomer-dependent, with approximately 17% of α-d-glucose and only 0.2% of β-d-glucose sampled in the ^1^C_4_ state. The larger ^1^C_4_ population for the α anomer is chemically reasonable because the axial anomeric hydroxyl group is expected to destabilize the ^4^C_1_ chair relative to β-d-glucose. Similar observations can be made for the oligomers (Figure S31). Thus, although limited sampling of the higher-energy chair is observed, the simulations do not show a collapse into an unphysical chair population that would explain the slow diffusion.

We also examined the hydroxymethyl rotamer distribution using the *ω* dihedral angle, defined as O5–C5–C6–O6 (Figure 8b). The *ω* states were assigned as gg for −120° < *ω* ≤ 0°, gt for 0° < *ω* ≤ 120°, and tg for *ω* ≤ −120° or *ω* > 120°. The resulting gg:gt ratio is approximately 27:71:2, indicating that gt is strongly favored, followed by gg, with only minor sampling of tg. This ordering agrees with the experimentally observed trend of gt > gg > tg for d-glucose.^77,78^ Therefore, the concentration-dependent slowdown in diffusion does not appear to arise from poor sampling of either the d-glucose chair conformation or the hydroxymethyl rotamer populations. Instead, it is more likely associated with overly strong water interactions at high concentration.

## Conclusion

This work evaluates Poltype2-generated AMOEBA parameters for d-glucose and 1→4-linked d-glucose oligomers, with an emphasis on how oxygen quadrupole scaling influences hydration properties, hydrogen bonding, conformational sampling, hydroxyl electrostatics, and translational diffusion. Because d-glucose contains multiple hydroxyl groups and forms extensive hydrogen-bonding networks with both water and neighboring d-glucose units, it provides a sensitive test case for assessing the transferability of polarizable electrostatic parameters behave from the monosaccharide to progressively larger 1→4-linked oligomers. Our results show that restoring hydroxyl oxygen quadrupoles to their full gas-phase values improves several aspects of d-glucose hydration. Relative to the recommended 0.7 oxygen quadrupole scaling, full 1.0 scaling shifts the monosaccharide hydration free energies toward commonly used experimental targets and gives an equilibrium-weighted hydration free energy of −26.6 kcal mol^–1^. The same scaling also increases d-glucose–water hydrogen-bond counts, with the strongest increase appearing in acceptor hydrogen bonds, and lengthens donor hydrogen-bond lifetimes. This donor/acceptor asymmetry suggests that oxygen quadrupole scaling not only strengthens the lone-pair acceptor region directly, but also modifies the electrostatic environment of the entire hydroxyl group. Importantly, these changes occur while the overall hydration-shell structure remains largely preserved.

In the 1→4-linked oligomers, oxygen quadrupole scaling also strengthens intramolecular hydrogen bonding and restricts conformational sampling. The 1.0-scaled β oligomers remain closer to their starting structures and retain a relatively linear geometry, while the α oligomers better preserve a fixed helical radius after converting from the initial left-handed helix to a favored right-handed conformation. Although the sampled glycosidic torsion distributions are comparable to those from other carbohydrate force fields, the offset between the simulated dominant α basin and experimental solution estimates suggests that the 1→4 glycosidic torsions may require further refinement independently of oxygen quadrupole scaling.

Electric-field analysis shows that increasing oxygen quadrupole scaling also enhances hydroxyl electrostatics, with projected fields exceeding 180 MV cm^–1^ at many sites. In monosaccharides, these large fields likely reflect electrostatic activation of solvent-exposed hydroxyls. In the 1→4-linked oligomers, however, large fields at OH2 and OH3, especially in the α system, arise mainly from d-glucose-derived intramolecular contributions associated with hydrogen-bond networks, making these hydroxyls more ordered but potentially less accessible. By contrast, OH6 remains largely water-controlled and solvent-exposed, consistent with its experimentally observed high reactivity.

Finally, diffusion simulations reveal both the strengths and limitations of the scaled models. At lower concentrations, both the 0.7- and 1.0-scaled models give diffusion coefficients close to experimental references, with the 1.0-scaled model slightly closer in the dilute limit. As concentration increases, however, the models diverge: the 1.0-scaled model predicts substantially slower diffusion than the 0.7-scaled model and both underestimate the experimental diffusion coefficient at 4.0 mol kg^–1^. Analysis of the d-glucose chair conformation and hydroxymethyl rotamer populations shows that this slowdown is not caused by poor sampling of the major intramolecular d-glucose conformations. Instead, it is more likely due to overly strong d-glucose–water and d-glucose–d-glucose interactions under concentrated solution conditions.

Taken together, these results show that full oxygen quadrupole scaling improves the description of local hydration, hydrogen bonding, and hydroxyl electrostatics in AMOEBA d-glucose models, and provides a more physically responsive description of carbohydrate hydroxyl groups. At the same time, the high-concentration diffusion results indicate that stronger local electrostatics must be balanced against collective transport properties. Note that these highly concentrated homogeneous solutions do not model locally crowded biological environments such as transporter cavities or protein tunnels. Distinct diagnostic tests are needed to validate the use of AMOEBA in these cases.

Future AMOEBA carbohydrate parameterization should therefore retain the improved local electrostatic description enabled by full oxygen quadrupole scaling while further refining intermolecular interaction balance and glycosidic torsional parameters for larger and more concentrated d-glucose systems. Extension to other monosaccharides and glycosidic linkage types, including 1→3 and 1→6 linkages, will be necessary to determine the generality of these observations.

## Supplementary Material



## Data Availability

The data supporting this article have been included as part of the Supplementary Information. Supporting Information includes RMSD and torsional potential energy analyses for α- and β-d-glucose oligomers; mean-square displacement plots for concentration-dependent diffusion calculations; hydrogen-bond lifetime autocorrelation functions; monosaccharide hydration free energy components; oligosaccharide hydration free energy fits and oxygen-normalized hydration free energy fits; site-resolved monosaccharide hydrogen-bond count analyses; d-glucose oxygen–water radial distribution functions and hydration-shell coordination numbers; per-oxygen oligomer–water and intramolecular hydrogen-bond count analyses; representative trajectory snapshots of α- and β-d-glucose 10-unit oligomers; individual glycosidic *ϕ*_H_/*ψ*_H_ distributions for oligosaccharides; and decomposed electric field projections for oligomers. Input files, including Poltype2 and Tinker9 input and parameter files, are available in our GitHub repository https://github.com/WelbornGroup/Newman_D-Glucose_2026/.

## Notes

RMSD and torsional potential-energy time series for α- and β-d-glucose oligosaccharides (Figures S1–S4). Mean-square displacement of aqueous d–glucose at different concentrations (Figure S5). Glucose–water hydrogen-bond lifetime autocorrelation functions and fit parameters (Figures S6–S7 and Table S1). Oligosaccharide hydration free-energy scaling and replicate-resolved monosaccharide free-energy contributions (Figure S8 and Table S2). Site-resolved monosaccharide hydrogen-bond counts and oxygen–water radial distribution functions (Figures S9–S11). Oligomer solvent-accessible surface area time series and chain-length dependence (Figures S12–S14). Site-resolved water–oligomer and intramolecular hydrogen-bond counts (Figures S15–S16). Trajectory snapshots of α- and β-d-glucose decamers under different quadrupole scaling conditions (Figures S17–S18 and S22–S23). Oligomer radius-of-gyration time series and chain-length dependence (Figures S19–S21). Glycosidic dihedral-angle distributions and comparison of calculated and experimental NMR J-coupling constants (Figures S24–S27 and Table S3). Water and intramolecular contributions to hydroxyl-bond electric fields (Figure S28). Anomer-resolved glucose diffusion coefficients versus concentration (Figure S29). Representative aqueous glucose configurations at different concentrations (Figure S30). Oligomerchair-conformation populations under different quadrupole scaling conditions (Figure S31) (PDF)
