# Supplementary material for: Refining AMOEBA Electrostatics for d‑Glucose and 1→4-Linked d‑Glucose Oligosaccharides
Source: J Chem Inf Model. 2026 Oct 2;66(20):13453–66. doi: 10.1021/acs.jcim.6c02832 (PMC13715523; doi:10.1021/acs.jcim.6c02832)
Supplement: Supplementary file 1 [file ci6c02832_si_001.pdf]

Refining AMOEBA Electrostatics for D-Glucose and 1→4-Linked D-Glucose Oligosaccharides Supporting Information

Luke A. Newman,[†,‡] Riley Ophof,[†] and Valerie Vaissier Welborn[*,†,‡]

†Department of Chemistry, Virginia Tech, Blacksburg, VA 24061, USA

‡Macromolecules Innovation Institute, Virginia Tech, Blacksburg, VA 24061, USA

E-mail: vwelborn@vt.edu

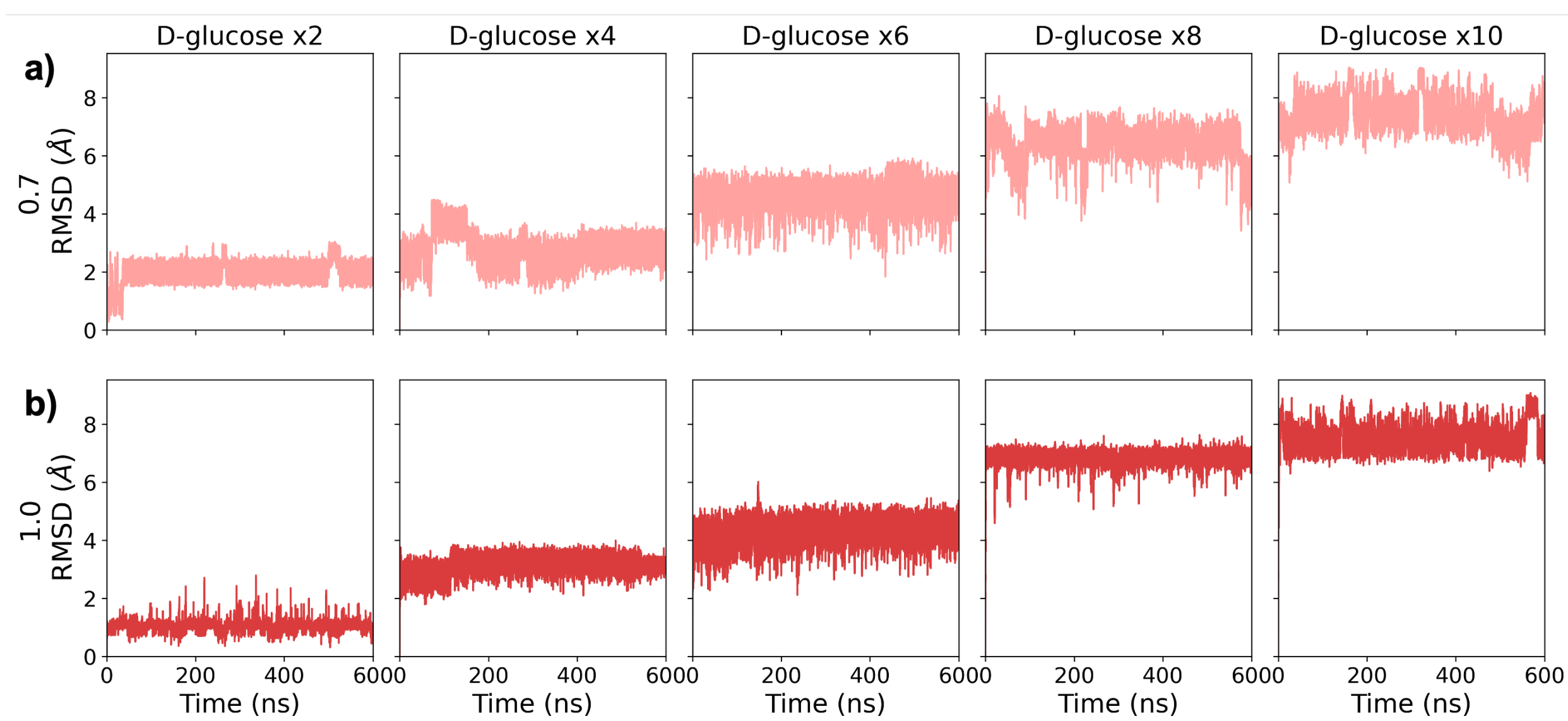

Figure S1: Root-mean-square deviation (RMSD) for α-D-glucose oligosaccharide systems under a) 0.7 and b) 1.0 oxygen quadrupole scaling.

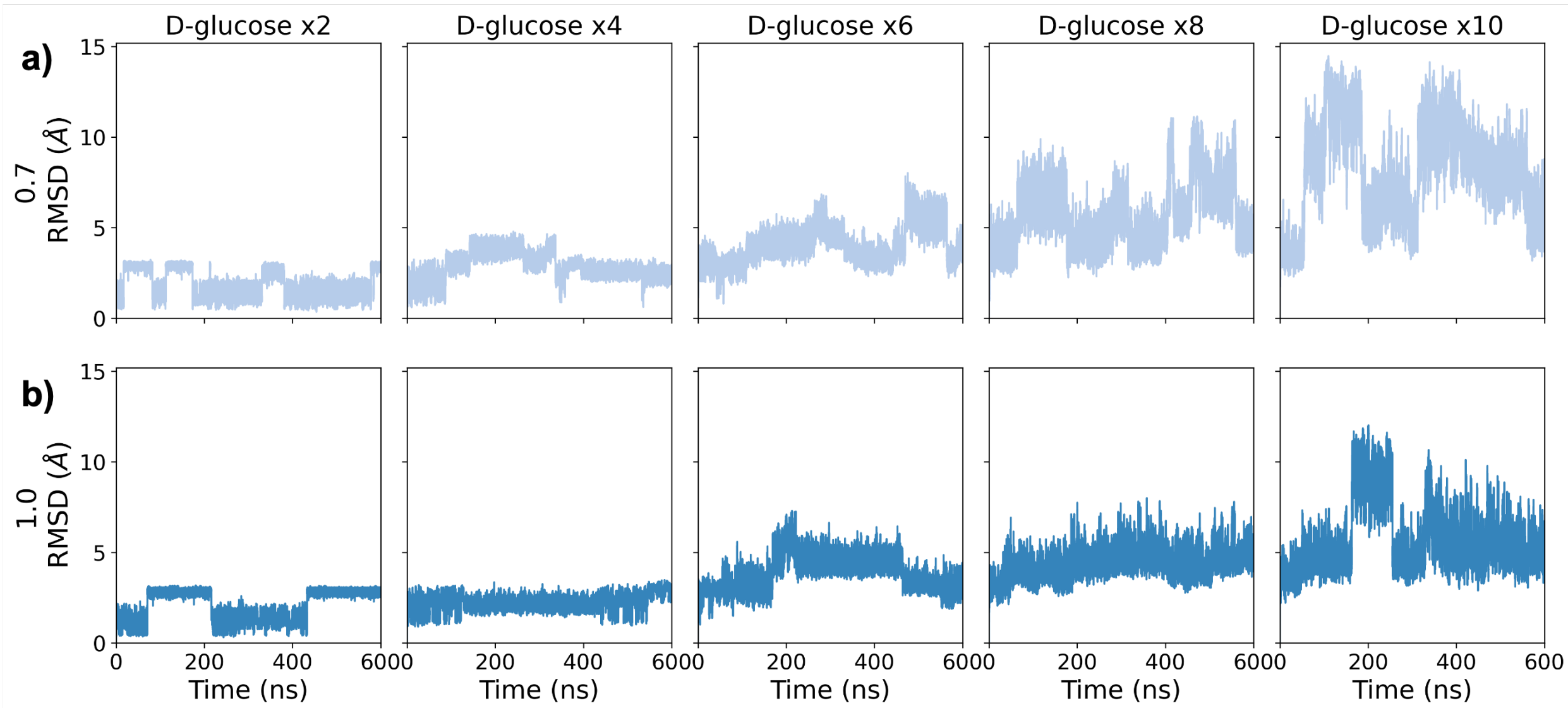

Figure S2: Root-mean-square deviation (RMSD) for β-D-glucose oligosaccharide systems under a) 0.7 and b) 1.0 oxygen quadrupole scaling.

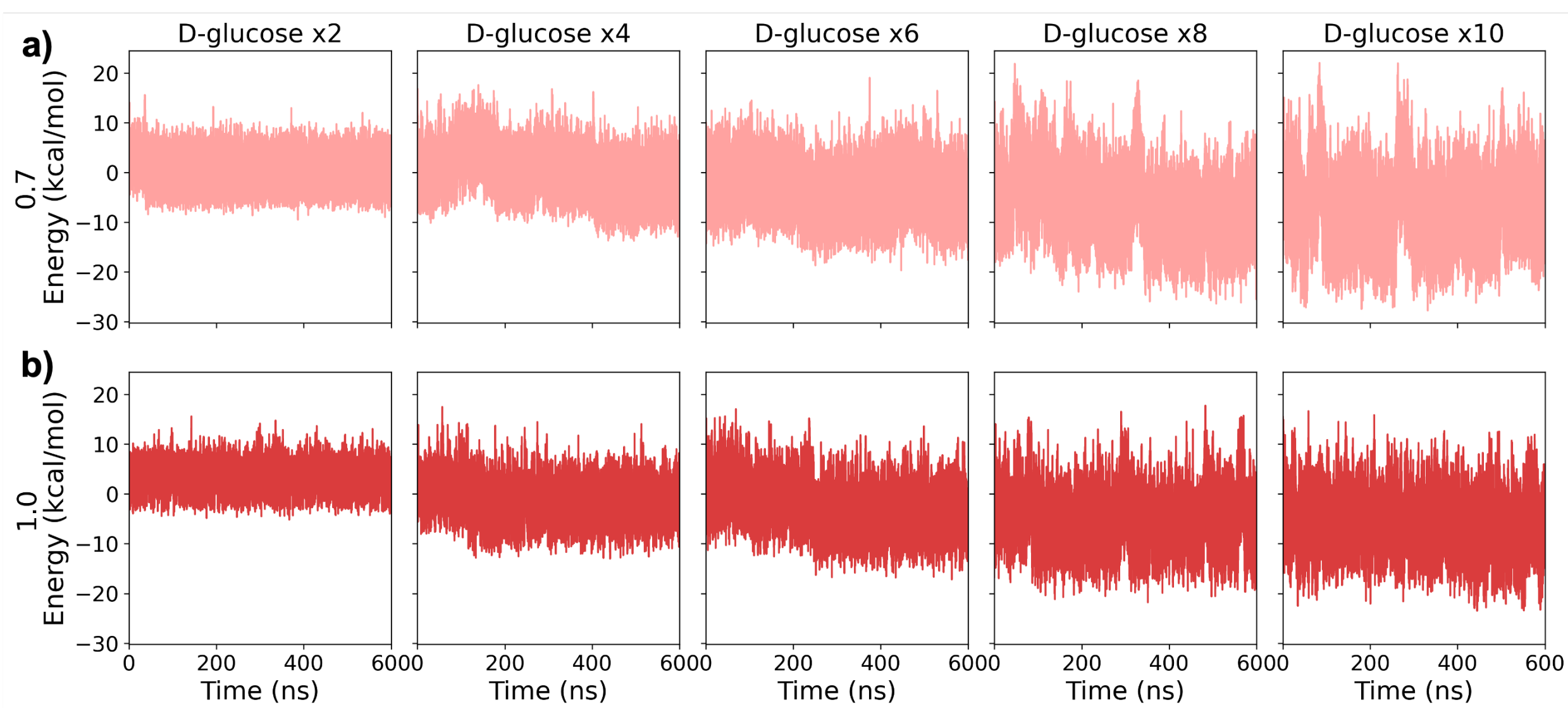

Figure S3: Torsional potential energy over time for α-D-glucose oligosaccharide systems under a) 0.7 and b) 1.0 oxygen quadrupole scaling.

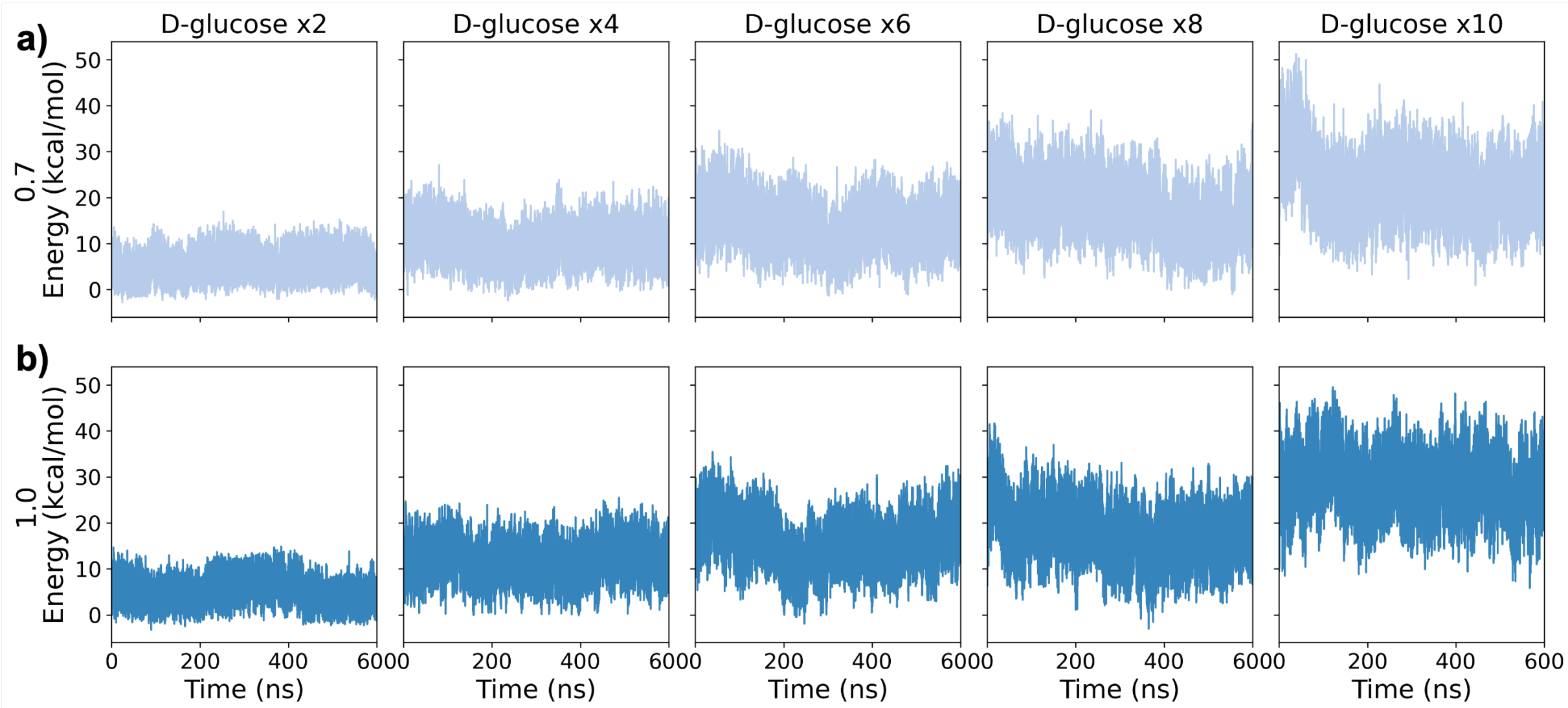

Figure S4: Torsional potential energy over time for β-D-glucose oligosaccharide systems under a) 0.7 and b) 1.0 oxygen quadrupole scaling.

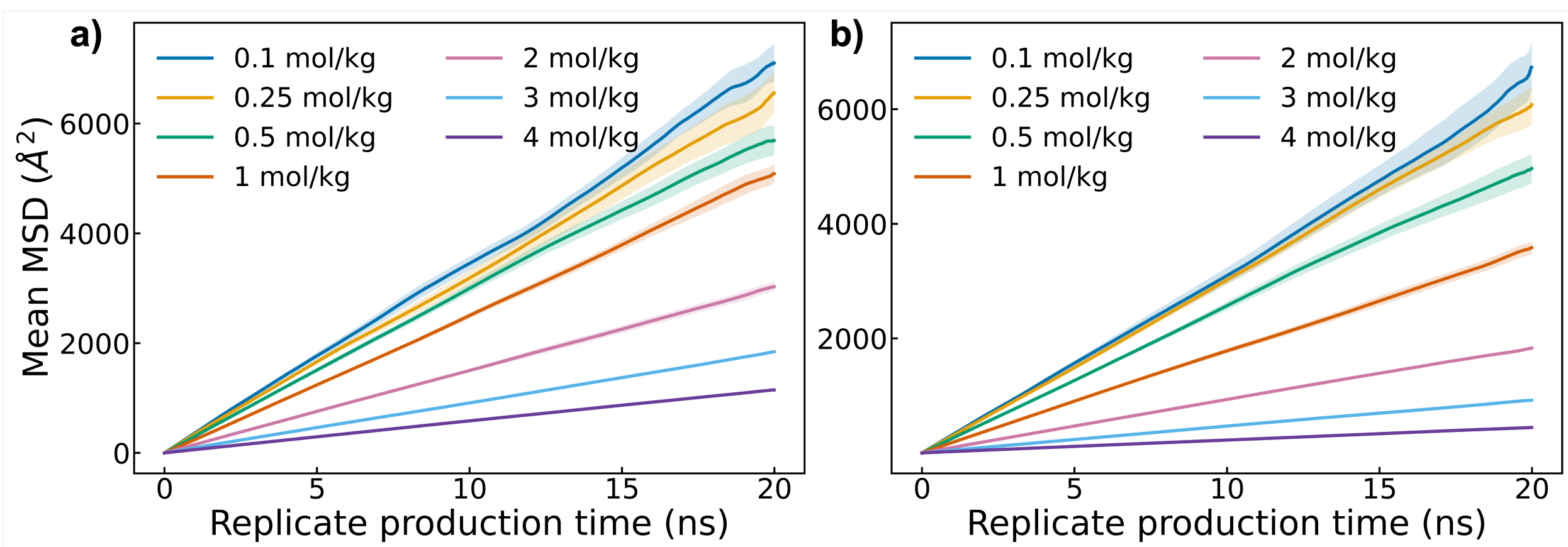

Figure S5: Replicate-averaged mean-square displacement (MSD) of the D-glucose center of mass in aqueous D-glucose systems. MSD curves are shown as a function of production time for each D-glucose concentration under a) 0.7 and b) 1.0 oxygen quadrupole scaling. Solid lines represent the mean across 10 independent 20 ns production simulations, and shaded regions represent the standard error of the mean.

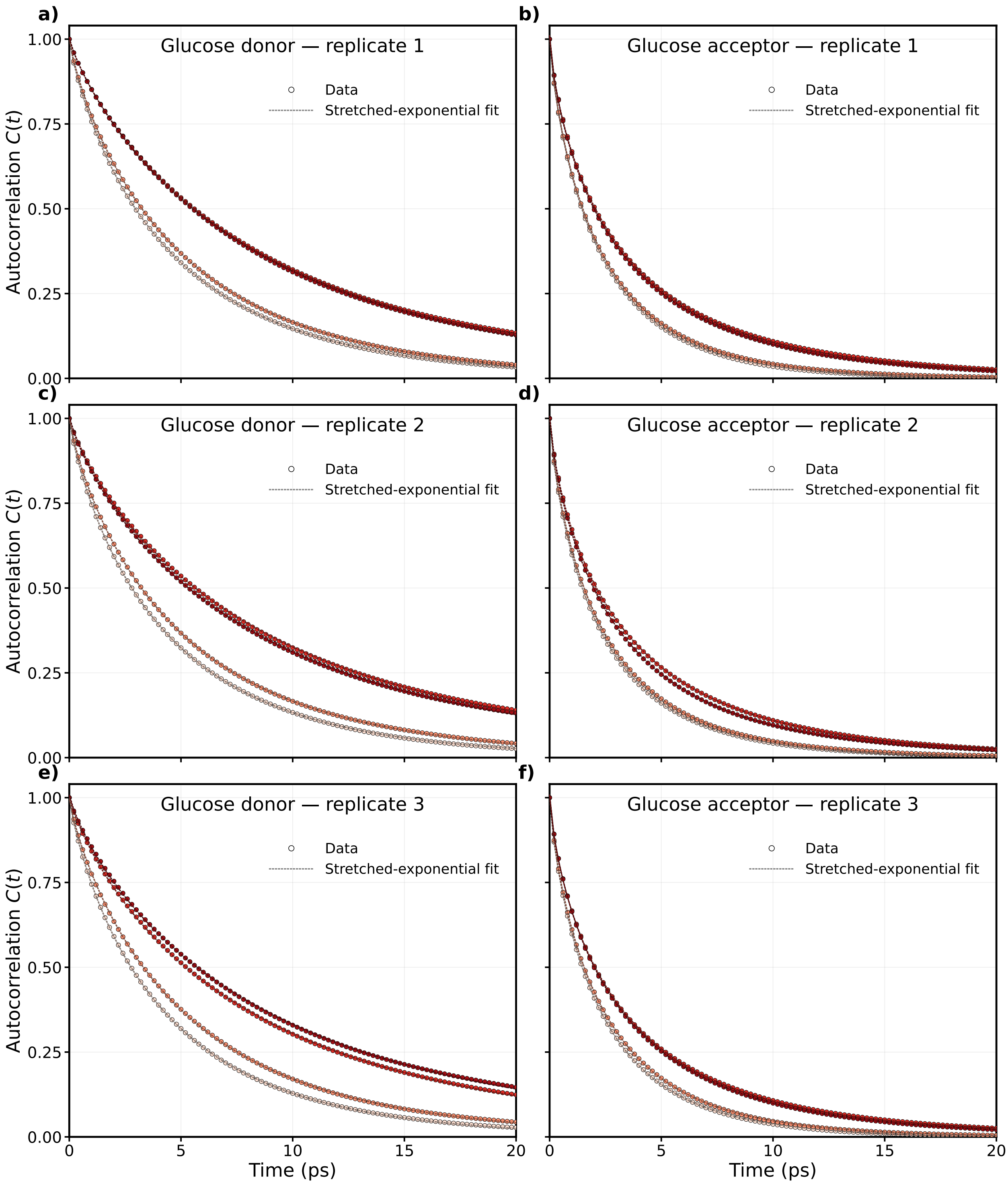

Figure S6: Hydrogen-bond lifetime autocorrelation functions for α-D-glucose–water hydrogen bonds across three replicate simulations. Panels a), c), and e) show α-D-glucose acting as a hydrogen-bond donor to water in replicates r1, r2, and r3, respectively; panels b), d), and f) show α-D-glucose acting as a hydrogen-bond acceptor from water in the corresponding replicates. Dashed lines represent stretched-exponential fits to the autocorrelation functions.

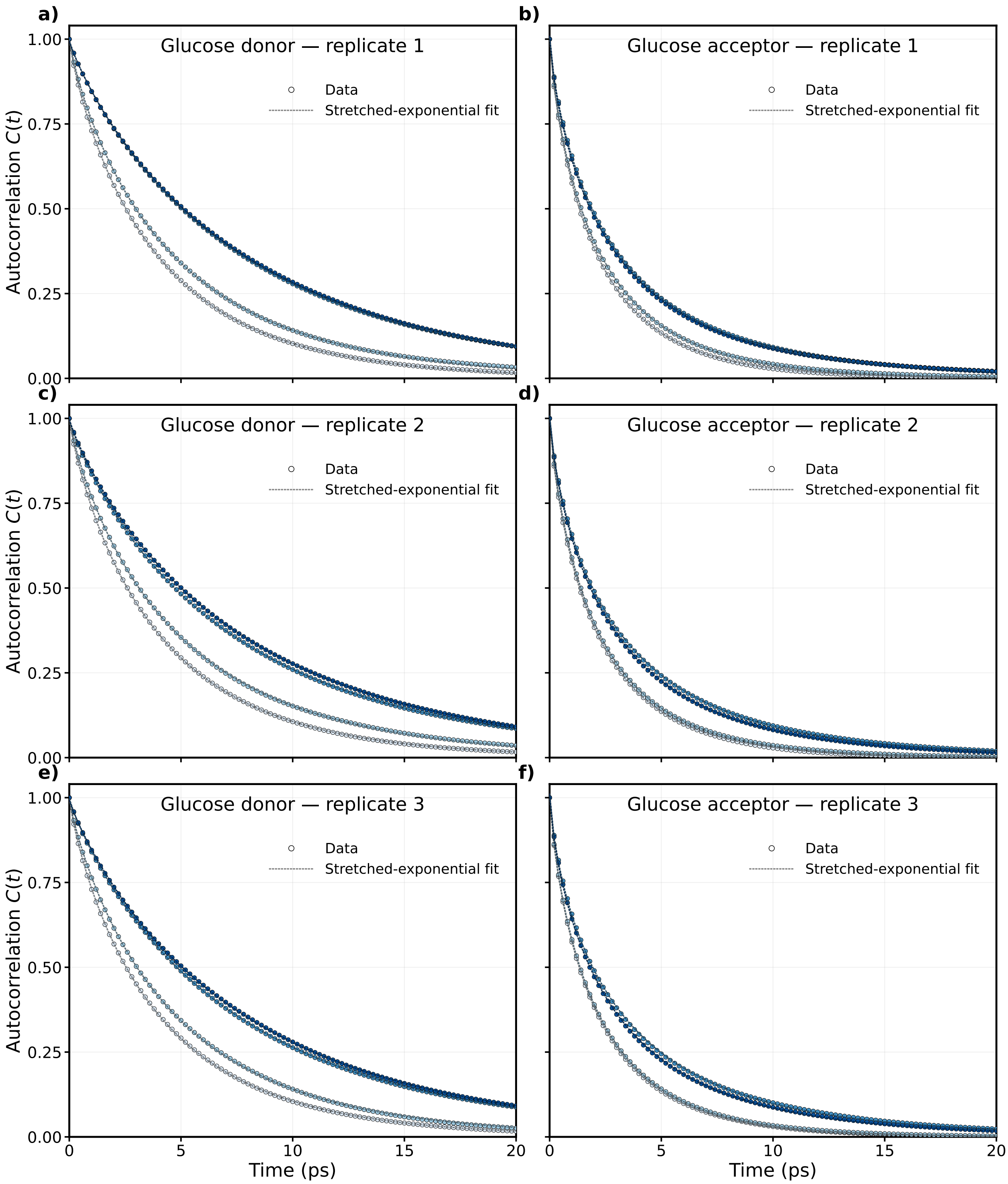

Figure S7: Hydrogen-bond lifetime autocorrelation functions for β-D-glucose–water hydrogen bonds across three replicate simulations. Panels a), c), and e) show β-D-glucose acting as a hydrogen-bond donor to water in replicates r1, r2, and r3, respectively; panels b), d), and f) show β-D-glucose acting as a hydrogen-bond acceptor from water in the corresponding replicates. Dashed lines represent stretched-exponential fits to the autocorrelation functions.

Table S1: Stretched-exponential fit parameters for the hydrogen-bond lifetime autocorrelation functions of α- and β-D-glucose with water. Here, we report the fitted decay timescale τ in ps, the stretching exponent β, and the coefficient of determination R^2. Values are reported separately for each replicate simulation.

			0.6_{OH}			0.7			1.0_{OH}			1.0		
**Anomer**	**Role**		τ **(ps)**	β	R^2	τ **(ps)**	β	R^2	τ **(ps)**	β	R^2	τ **(ps)**	β	R^2
α-D-glucose	Donor	r1	4.59	0.839	0.999997	5.02	0.849	0.999995	8.59	0.844	0.999966	8.44	0.852	0.999965
		r2	4.34	0.841	0.999994	4.99	0.838	0.999989	8.69	0.832	0.999925	8.26	0.823	0.999948
		r3	4.27	0.844	0.999983	5.13	0.849	0.999979	8.07	0.827	0.999950	8.79	0.823	0.999868
	Acceptor	r1	2.27	0.808	0.999979	2.35	0.793	0.999995	3.31	0.728	0.999888	3.21	0.739	0.999941
		r2	2.31	0.786	0.999991	2.45	0.789	0.999993	3.40	0.739	0.999969	3.16	0.747	0.999895
		r3	2.30	0.804	0.999979	2.45	0.793	0.999975	3.30	0.731	0.999930	3.23	0.745	0.999969
β-D-glucose	Donor	r1	3.90	0.868	0.999981	4.58	0.855	0.999969	7.63	0.895	0.999992	7.75	0.897	0.999980
		r2	3.98	0.873	0.999984	4.79	0.855	0.999962	7.16	0.883	0.999986	7.58	0.894	0.999995
		r3	3.92	0.864	0.999979	4.65	0.875	0.999953	7.28	0.892	0.999971	7.67	0.904	0.999967
	Acceptor	r1	2.10	0.805	0.999988	2.26	0.783	0.999968	3.06	0.745	0.999844	2.94	0.736	0.999808
		r2	2.11	0.802	0.999929	2.21	0.801	0.999989	3.11	0.743	0.999928	2.93	0.754	0.999955
		r3	2.09	0.799	0.999995	2.15	0.797	0.999996	3.12	0.726	0.999836	2.91	0.733	0.999866

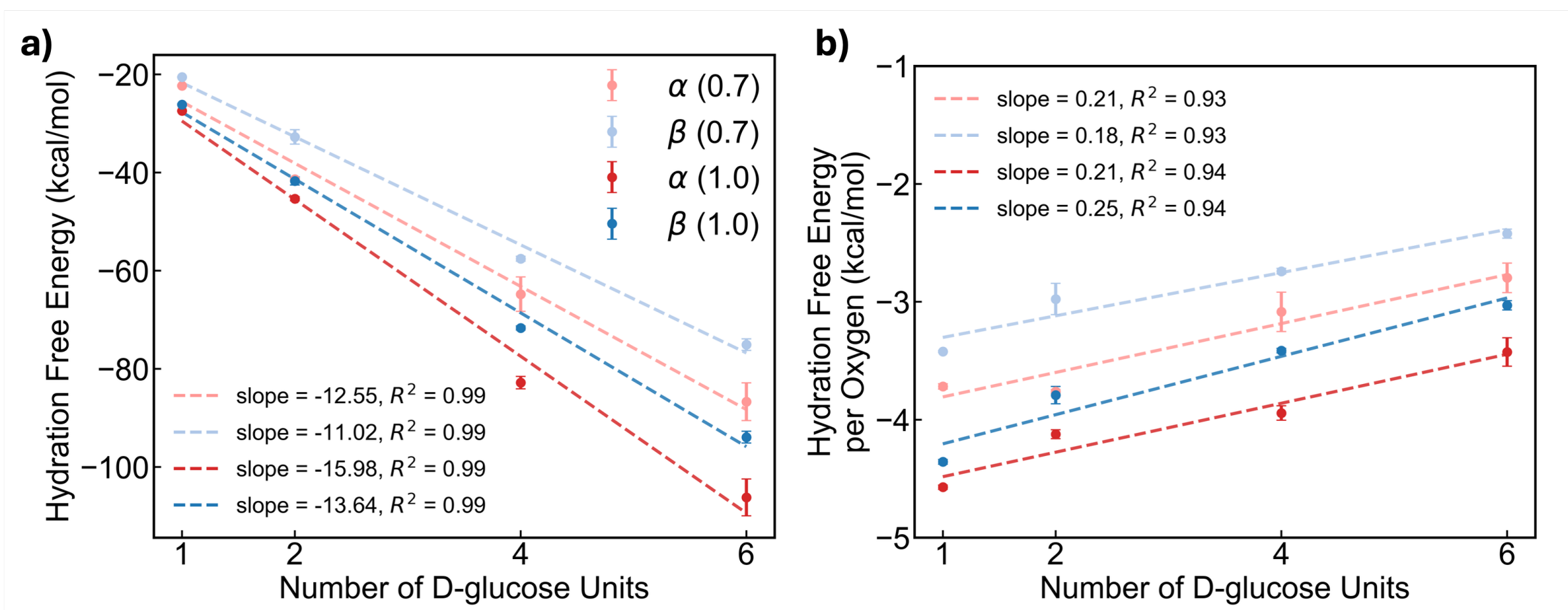

Figure S8: a) Linear fits of hydration free energy for α- and β-D-glucose oligosaccharides as a function of chain length. b) Linear fits of hydration free energy normalized by the number of oxygen atoms in each oligosaccharide.

Table S2: Individual monosaccharide hydration free-energy contributions for each replicate. All values are reported in kcal/mol.

**Anomer**	**Scaling**	**Replicate**	**HFE**	$\Delta G_{\mathrm{ele}}^{\mathrm{sol}}$	$\Delta G_{\mathrm{vdW}}^{\mathrm{sol}}$	$\Delta G_{\mathrm{ele}}^{\mathrm{vac}}$
α-D-glucose	0.6_{OH}	r1	-20.80	25.05	-4.47	-0.22
		r2	-21.05	25.19	-4.51	-0.37
		r3	-20.96	25.00	-4.52	-0.48
	0.7	r1	-22.28	26.43	-4.45	-0.30
		r2	-22.39	26.39	-4.52	-0.53
		r3	-22.25	26.35	-4.46	-0.36
	1.0_{OH}	r1	-27.28	31.64	-4.49	-0.13
		r2	-27.52	31.68	-4.47	-0.30
		r3	-27.56	31.92	-4.53	-0.17
	1.0	r1	-27.32	31.79	-4.63	-0.16
		r2	-27.60	31.74	-4.47	-0.32
		r3	-27.38	31.72	-4.50	-0.17
β-D-glucose	0.6_{OH}	r1	-18.96	23.14	-4.60	-0.42
		r2	-19.09	23.06	-4.55	-0.58
		r3	-19.22	23.13	-4.56	-0.65
	0.7	r1	-20.51	24.34	-4.61	-0.78
		r2	-20.56	24.32	-4.52	-0.77
		r3	-20.51	24.32	-4.60	-0.80
	1.0_{OH}	r1	-25.87	29.27	-4.50	-1.11
		r2	-25.98	29.30	-4.64	-1.32
		r3	-26.08	29.36	-4.58	-1.29
	1.0	r1	-26.18	29.34	-4.54	-1.37
		r2	-26.20	29.31	-4.54	-1.43
		r3	-26.05	29.26	-4.66	-1.45

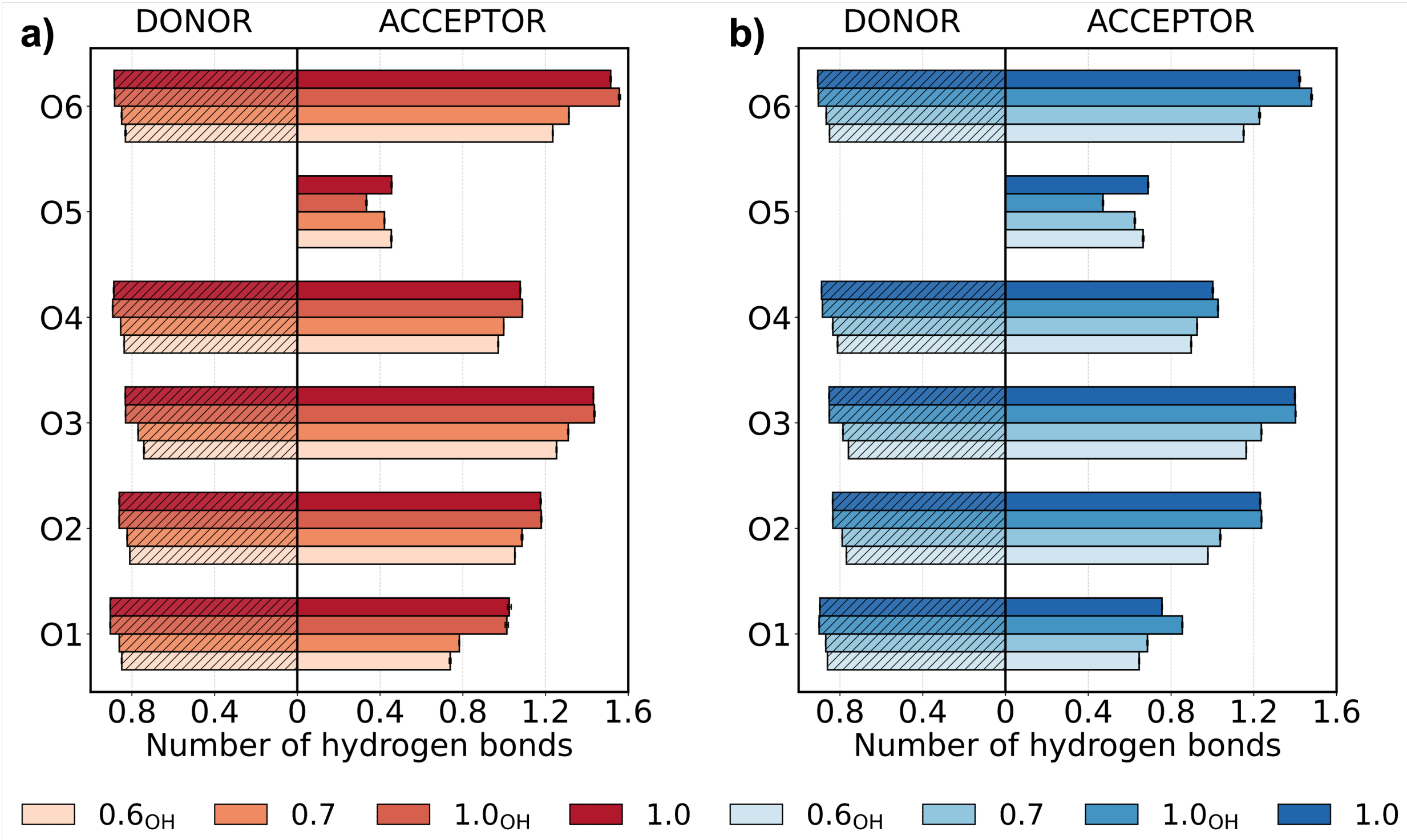

Figure S9: Average donor and acceptor hydrogen-bond counts by oxygen site for a) α-D-glucose and b) β-D-glucose monosaccharides. Hatched bars extending left show donor contributions from hydroxyl groups, and solid bars extending right show acceptor contributions at oxygen atoms. Within each site, quadrupole scaling values are ordered from top to bottom as 1.0, 1.0_{OH}, 0.7, and 0.6_{OH}. Error bars represent the standard error of the mean from 90 ns trajectory blocks separated by 10 ns gaps.

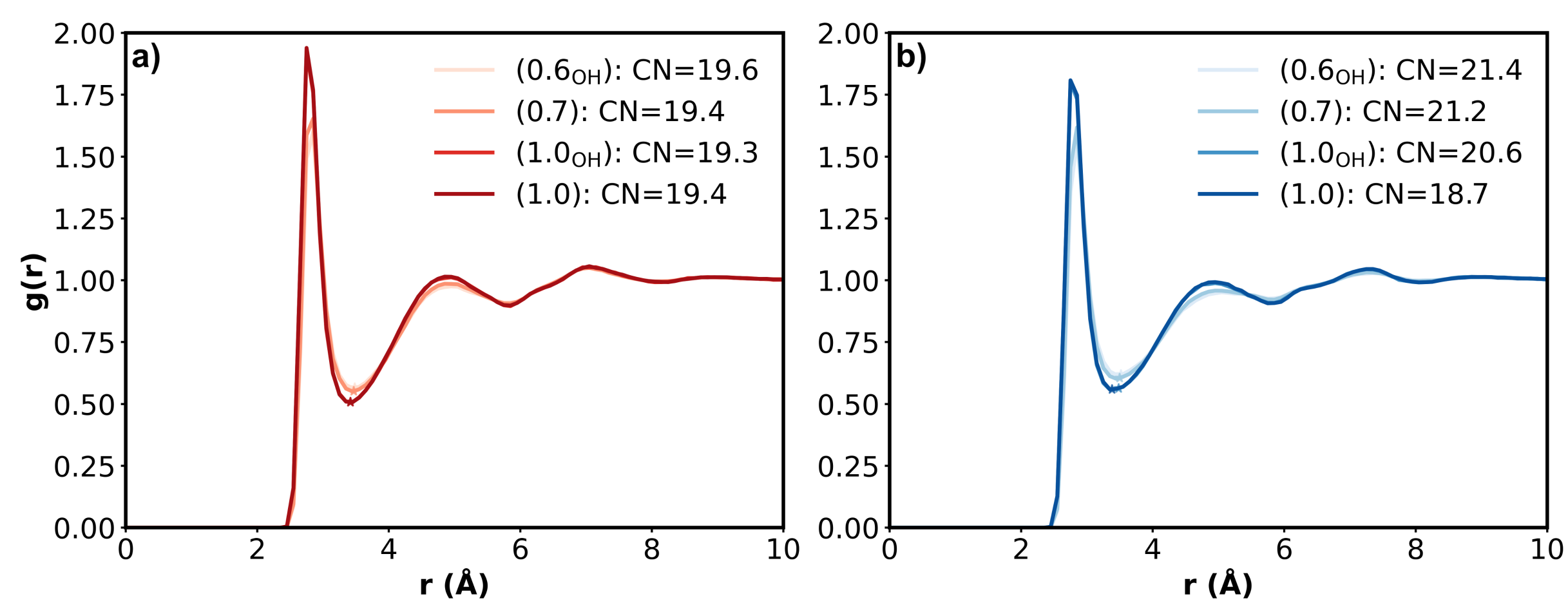

Figure S10: Oxygen–water radial distribution functions (RDFs) averaged over all oxygen atoms in a) α-D-glucose and b) β-D-glucose monosaccharides under different oxygen quadrupole scaling conditions. Coordination numbers are reported in the legend and were obtained by integrating each RDF to the first minimum, indicated by a star.

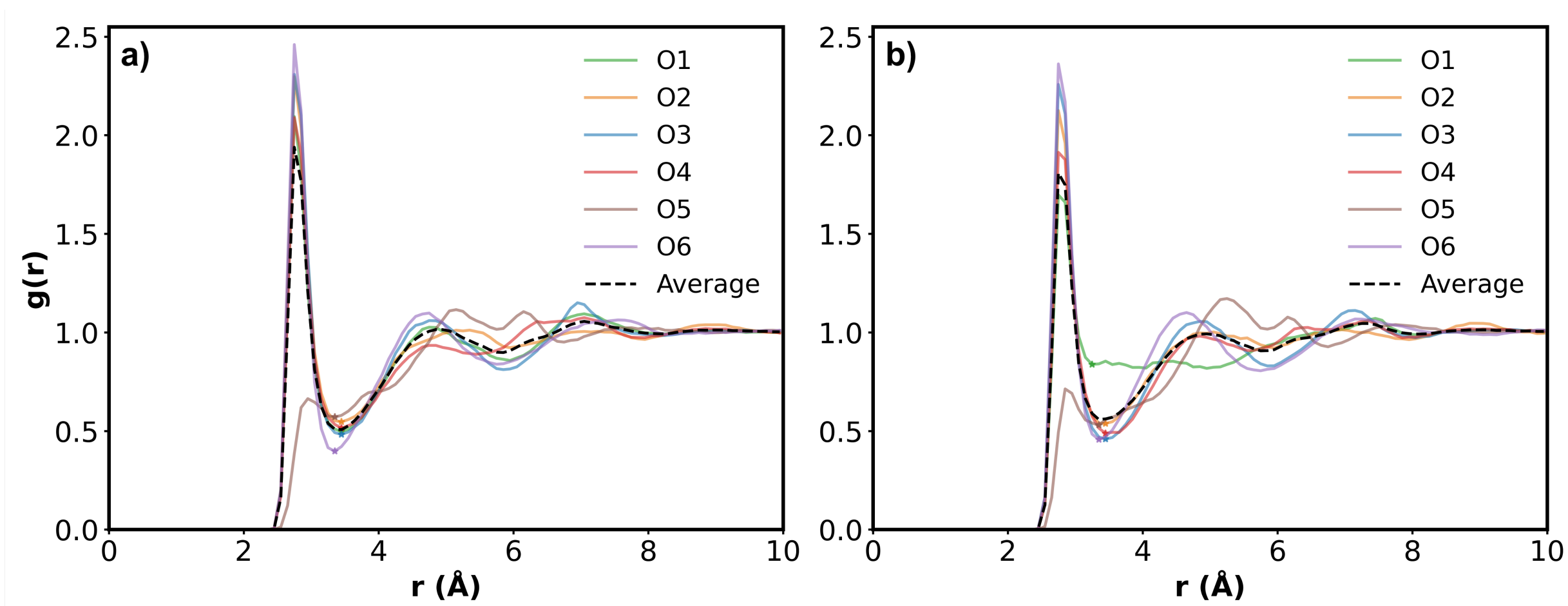

Figure S11: Site-resolved oxygen–water radial distribution functions (RDFs) for individual oxygen atoms in the full 1.0 oxygen quadrupole scaling models of a) α-D-glucose and b) β-D-glucose monosaccharides.

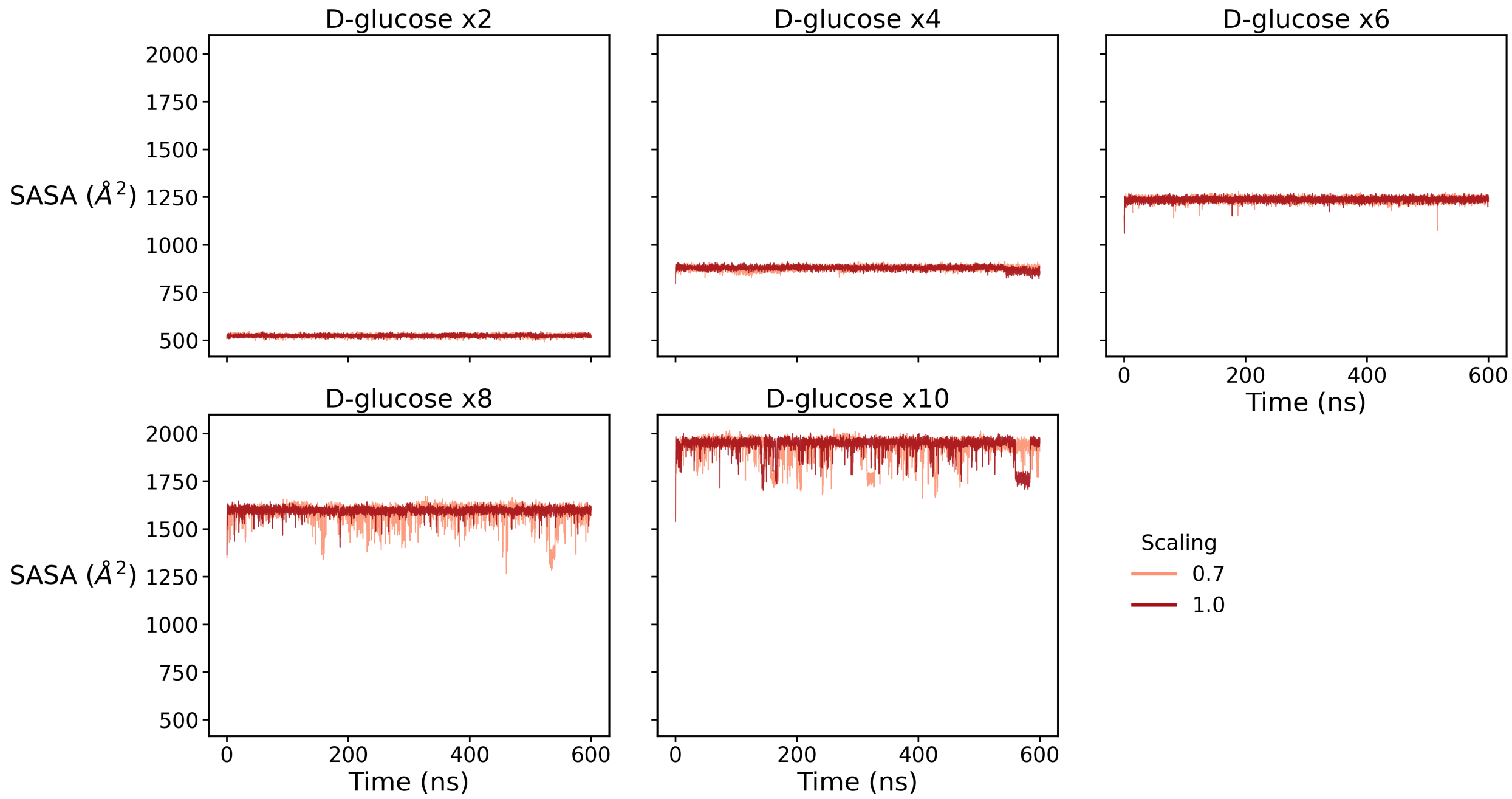

Figure S12: Solvent-accessible surface-area time series for α-D-glucose oligomers at oxygen quadrupole scaling factors of 0.7 and 1.0. Panels correspond to oligomers containing 2, 4, 6, 8, and 10 D-glucose units.

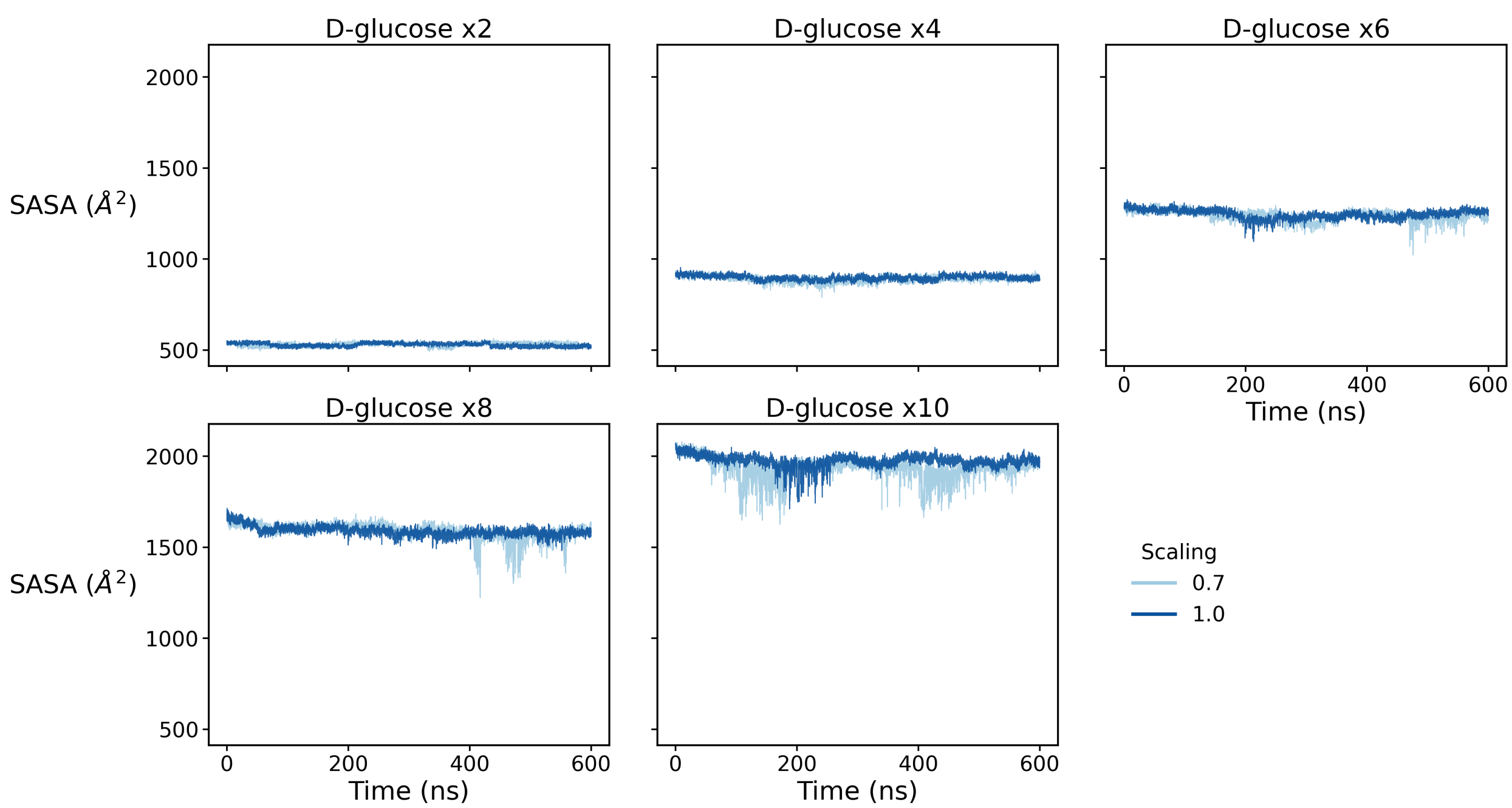

Figure S13: Solvent-accessible surface-area time series for β-D-glucose oligomers at oxygen quadrupole scaling factors of 0.7 and 1.0. Panels correspond to oligomers containing 2, 4, 6, 8, and 10 D-glucose units.

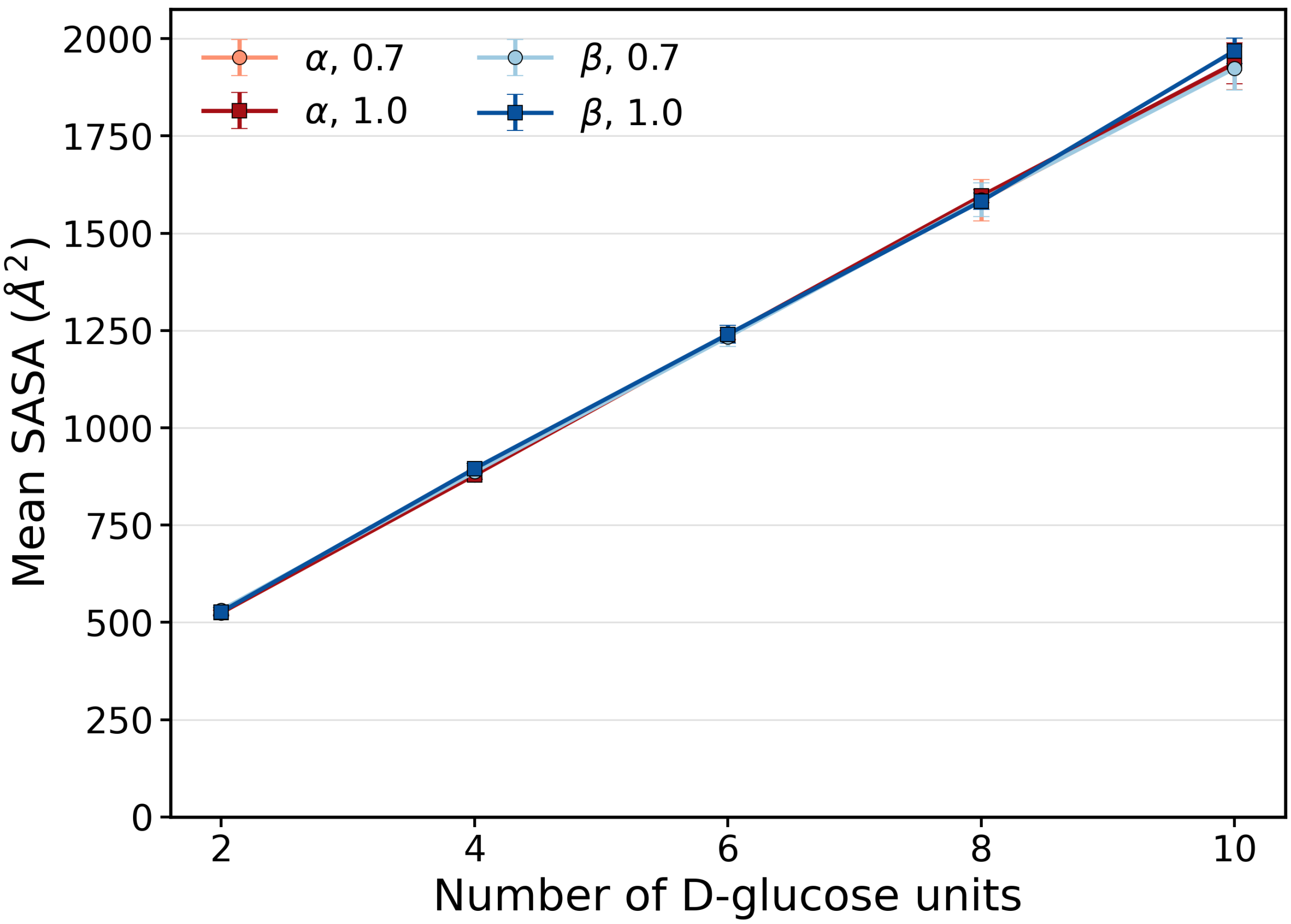

Figure S14: Mean solvent-accessible surface area as a function of oligomer length for α- and β-D-glucose oligomers at oxygen quadrupole scaling factors of 0.7 and 1.0. Error bars represent the standard deviation over trajectory frames.

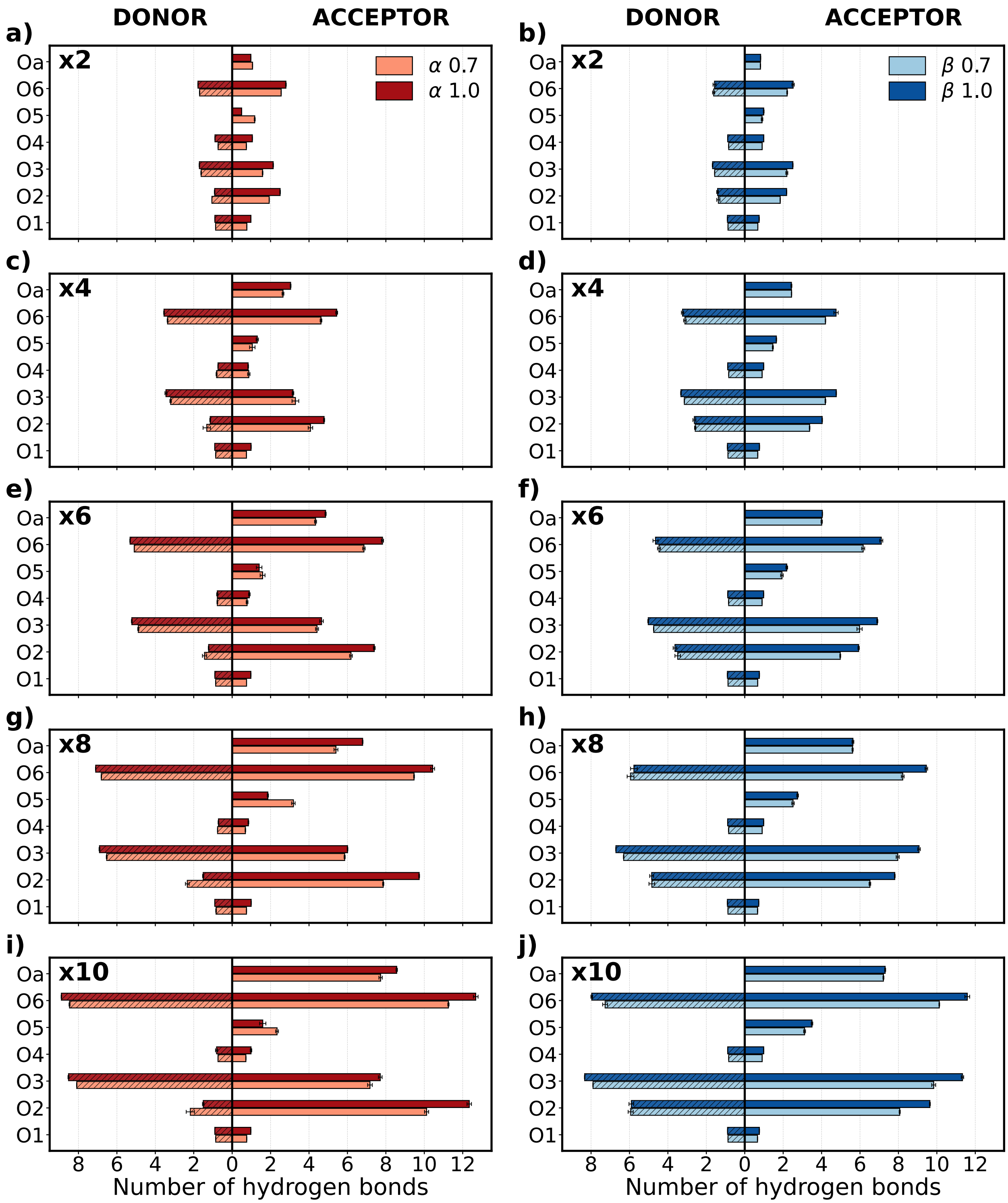

Figure S15: Per-oxygen water–oligomer hydrogen-bond counts for α- and β-D-glucose oligomers at 0.7 and 1.0 oxygen quadrupole scaling. Donor contributions are shown on the left and acceptor contributions on the right for each oxygen site. Panels correspond to increasing oligomer length from x2 to x10 D-glucose units, with error bars representing SEM values from 90 ns block averages separated by 10 ns gaps.

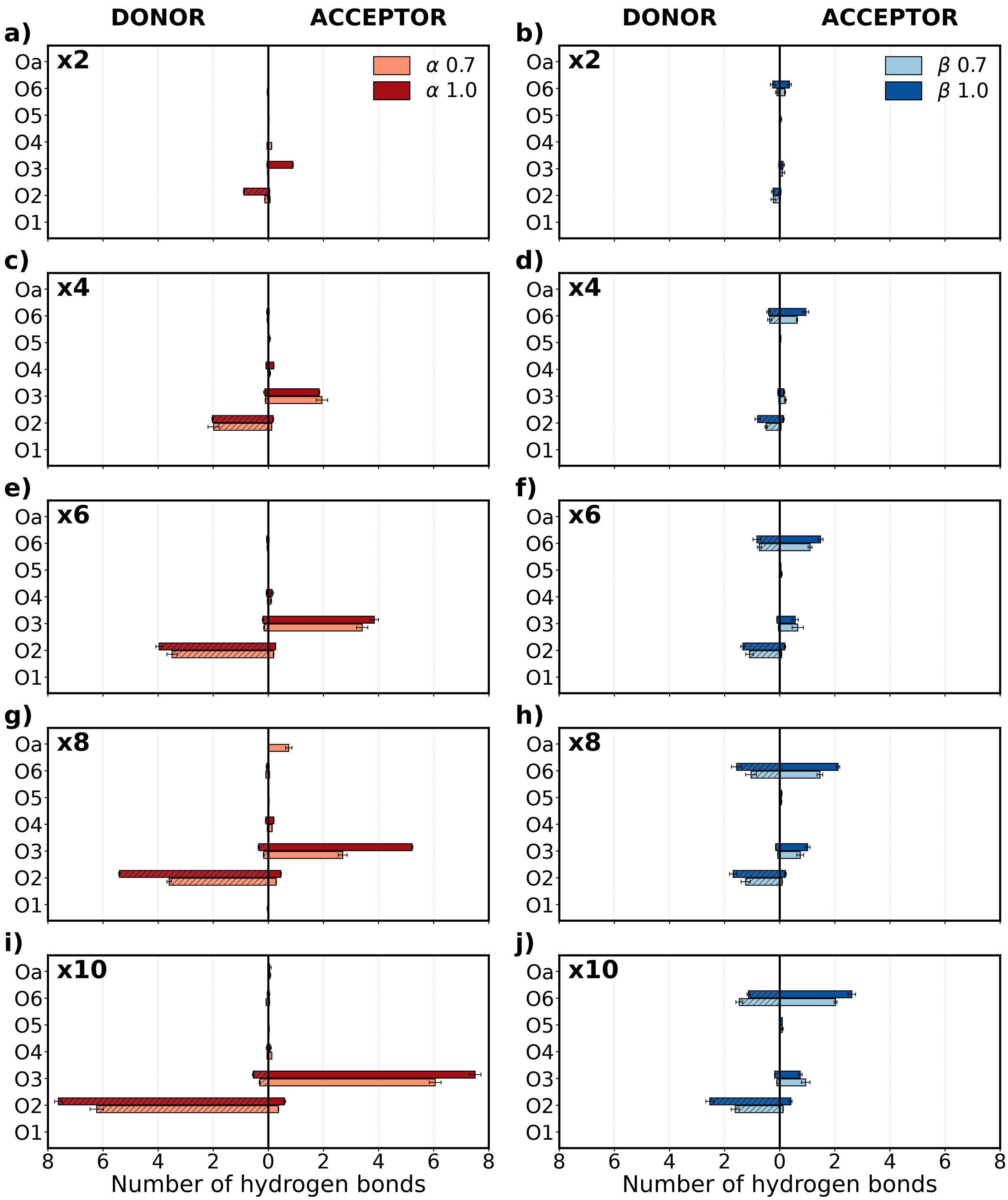

Figure S16: Per-oxygen intramolecular, oligomer–oligomer, hydrogen-bond counts for α- and β-D-glucose oligomers at 0.7 and 1.0 oxygen quadrupole scaling. Donor contributions are shown on the left and acceptor contributions on the right for each oxygen site. Panels correspond to increasing oligomer length from x2 to x10 D-glucose units, with error bars representing SEM values from 90 ns block averages separated by 10 ns gaps.

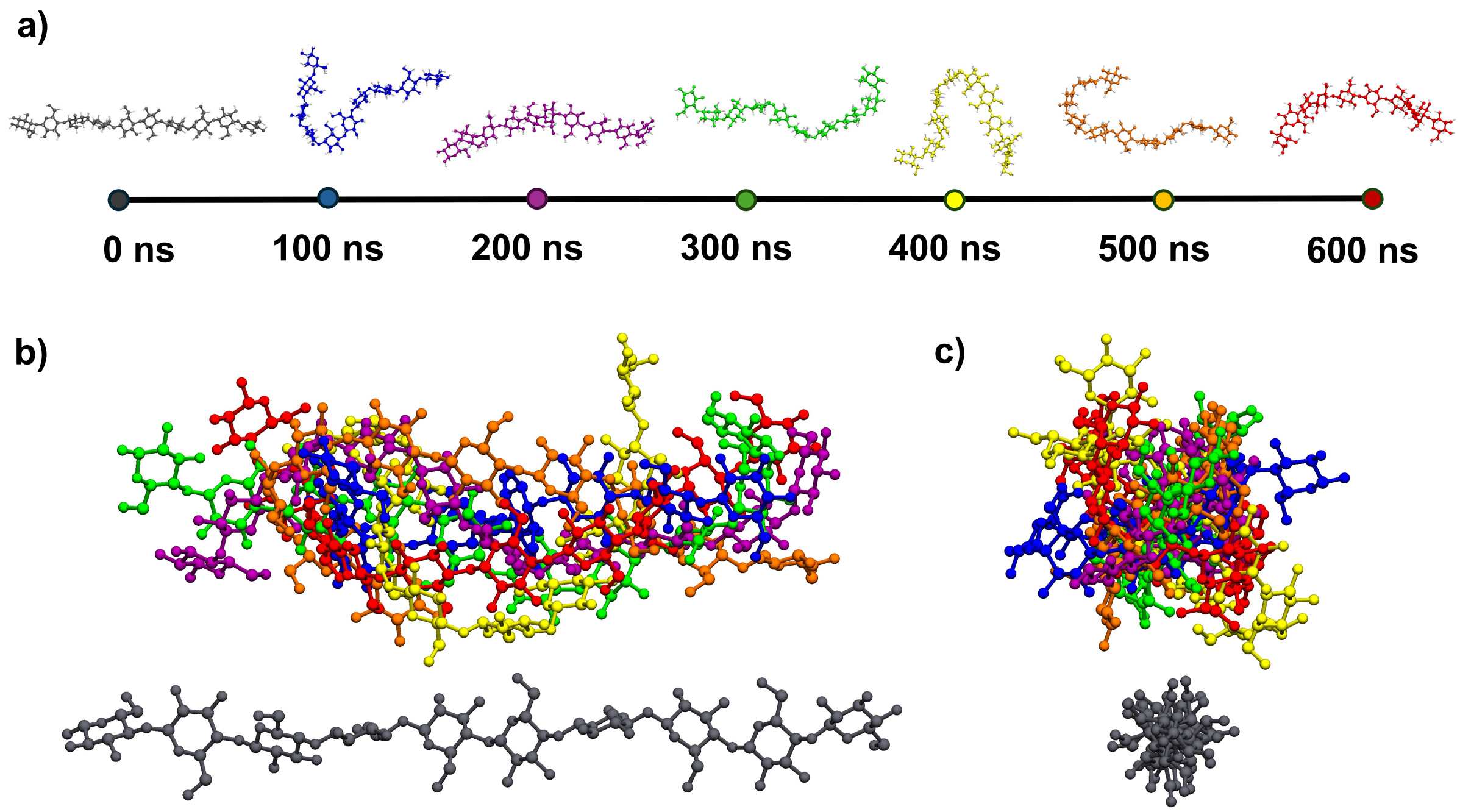

Figure S17: Representative trajectory snapshots of the 0.7-scaled β-D-glucose 10-unit oligomer. (a) Snapshot structures shown at 100 ns increments. All trajectory snapshots were aligned to the 0 ns starting structure, which is shown in gray for reference. Aligned snapshots are shown from (b) the side view and (c) down the length of the oligomer.

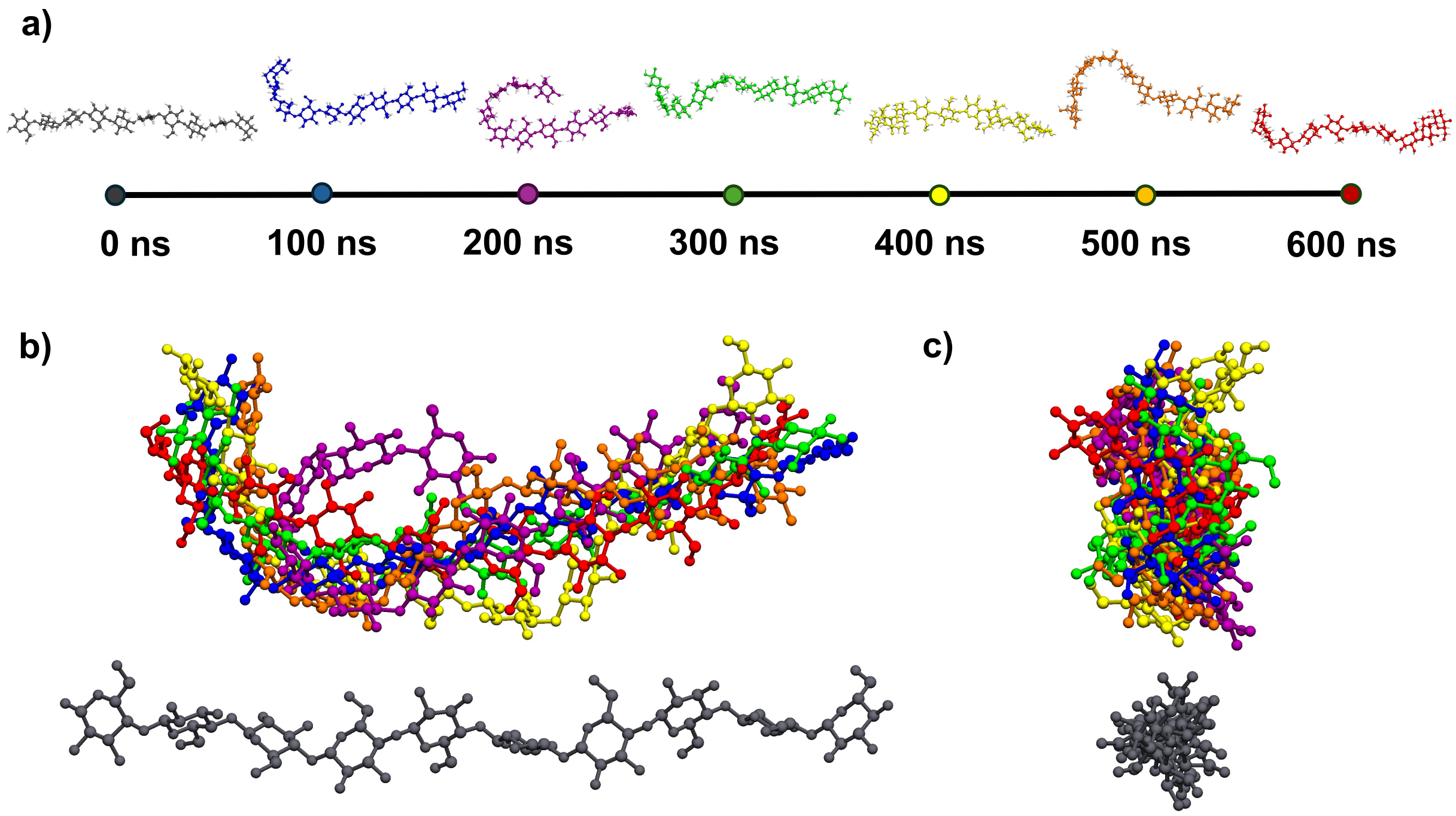

Figure S18: Representative trajectory snapshots of the 1.0-scaled β-D-glucose 10-unit oligomer. (a) Snapshot structures shown at 100 ns increments. All trajectory snapshots were aligned to the 0 ns starting structure, which is shown in gray for reference. Aligned snapshots are shown from (b) the side view and (c) down the length of the oligomer.

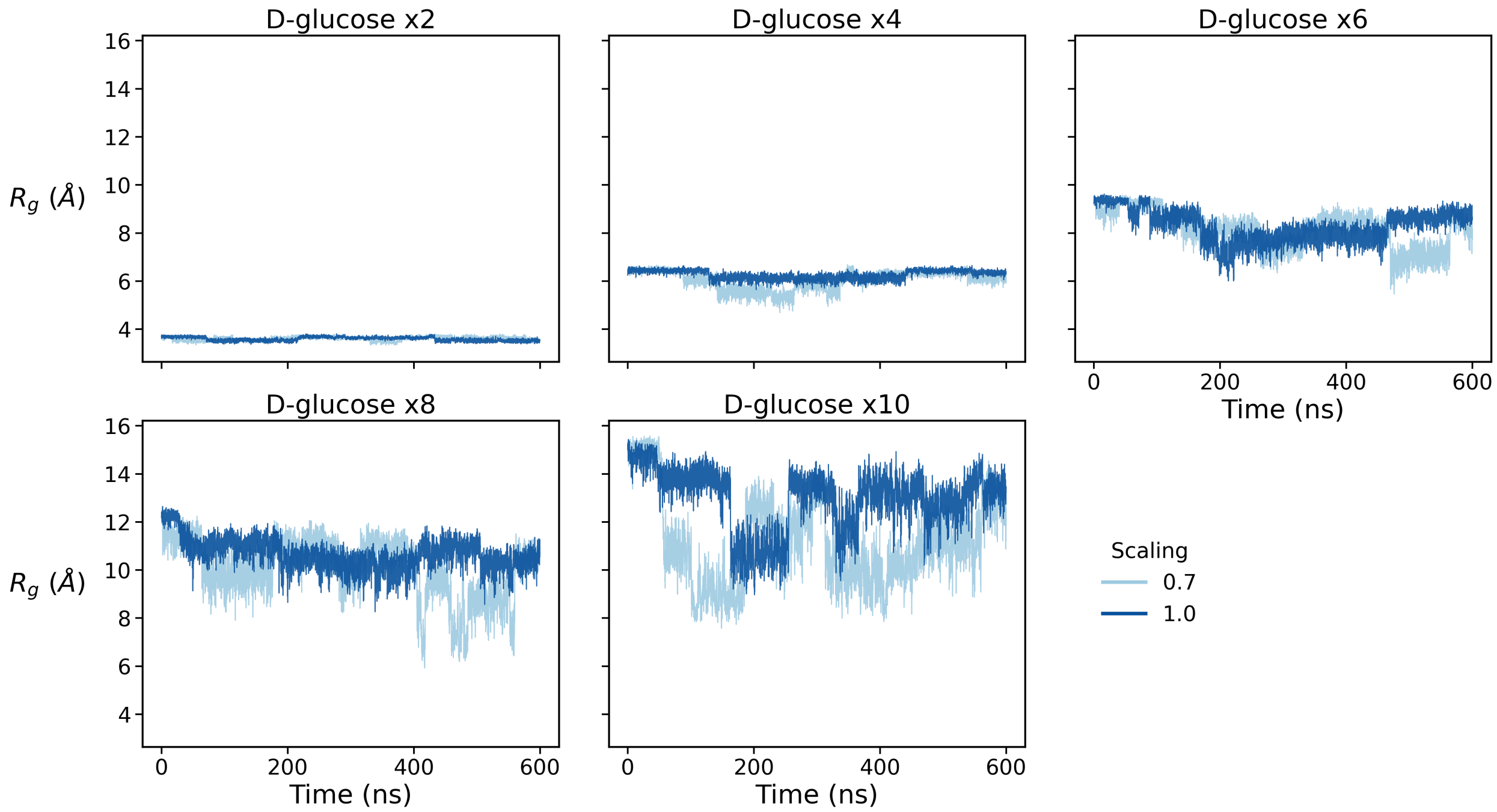

Figure S19: Mass-weighted radius-of-gyration time series for β-D-glucose oligomers at oxygen quadrupole scaling factors of 0.7 and 1.0. Panels correspond to oligomers containing 2, 4, 6, 8, and 10 D-glucose units.

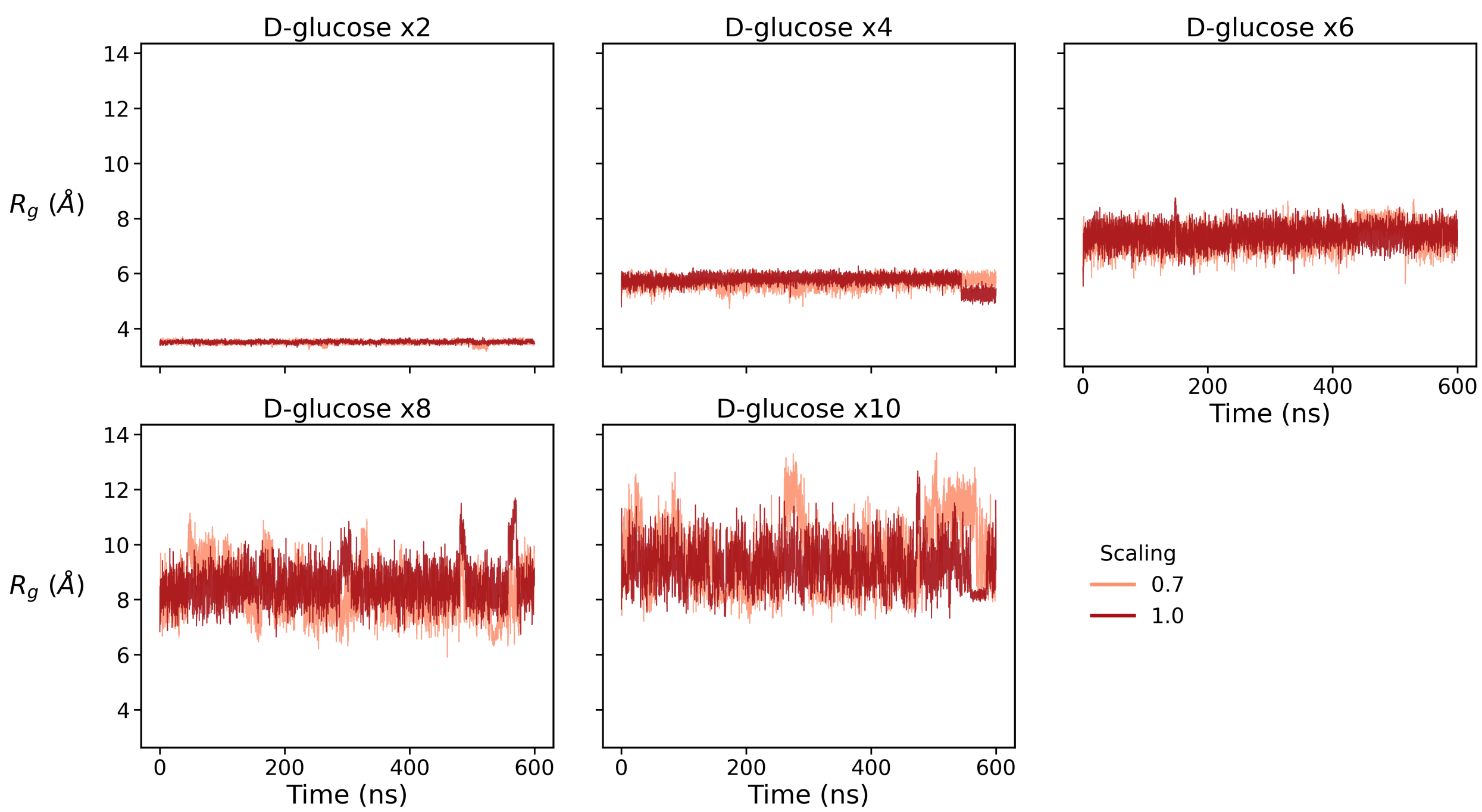

Figure S20: Mass-weighted radius-of-gyration time series for α-D-glucose oligomers at oxygen quadrupole scaling factors of 0.7 and 1.0. Panels correspond to oligomers containing 2, 4, 6, 8, and 10 D-glucose units.

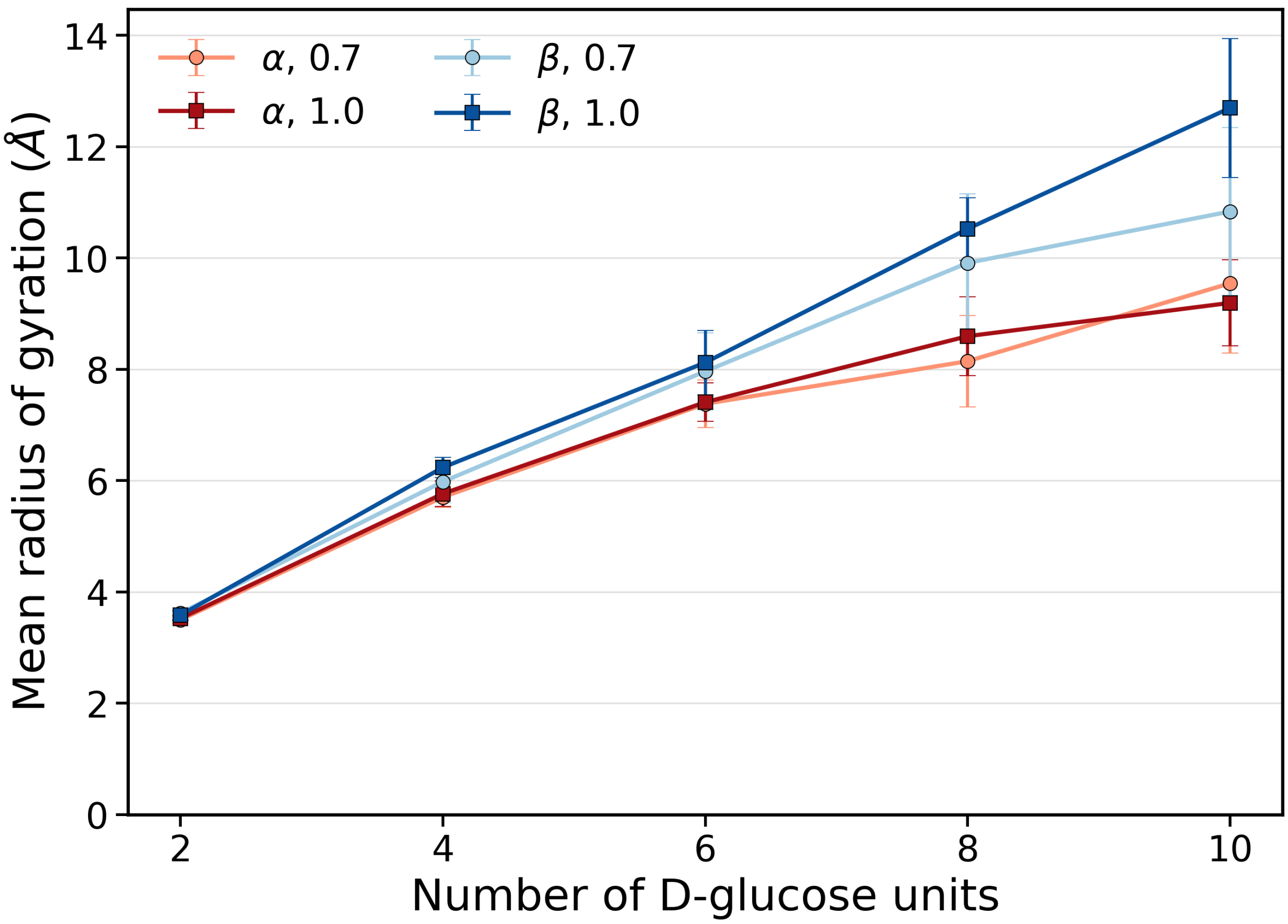

Figure S21: Mean mass-weighted radius of gyration as a function of oligomer length for α- and β-D-glucose oligomers at oxygen quadrupole scaling factors of 0.7 and 1.0. Error bars represent the standard deviation over trajectory frames.

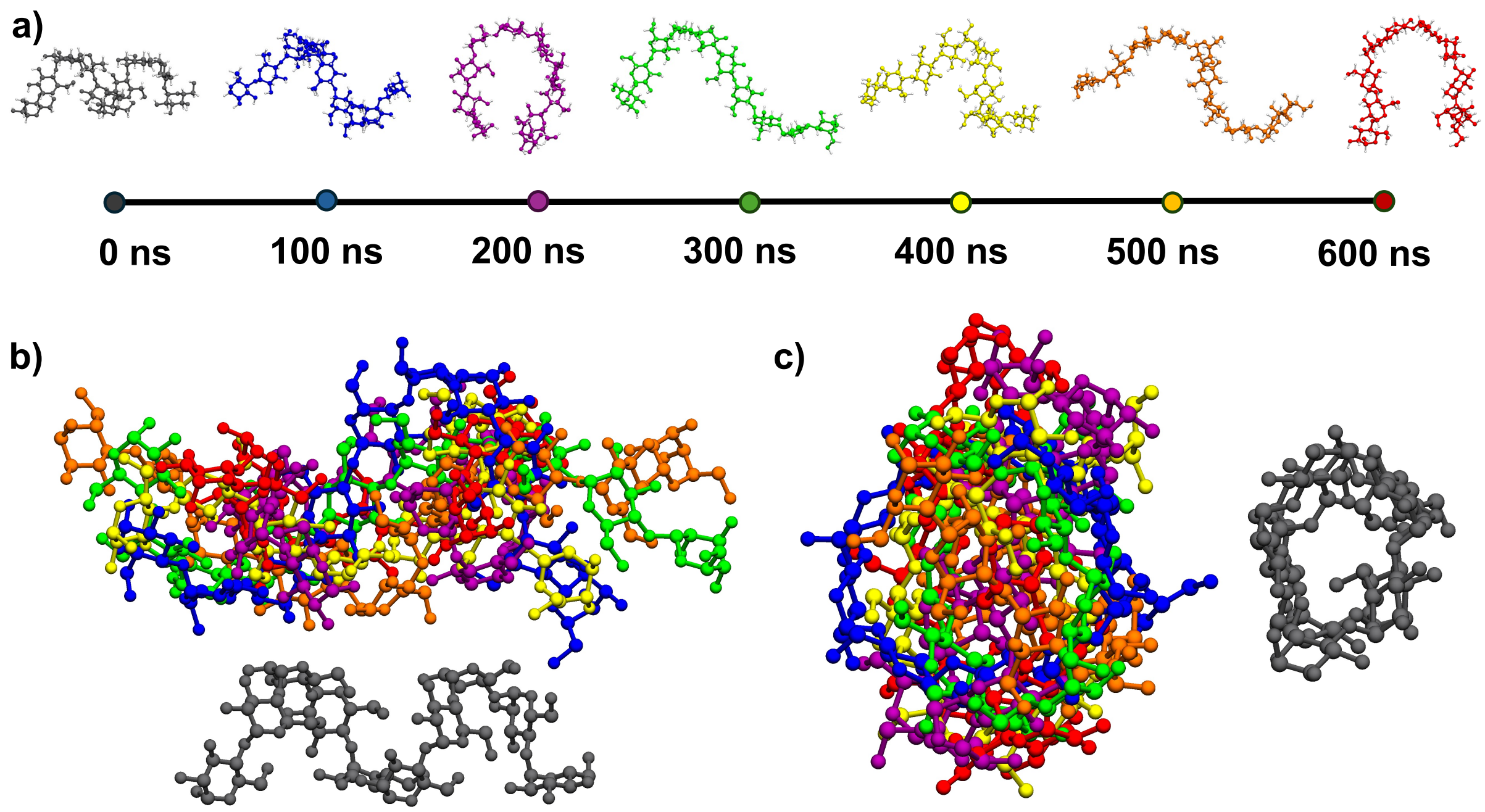

Figure S22: Representative trajectory snapshots of the 0.7-scaled α-D-glucose 10-unit oligomer. (a) Snapshot structures shown at 100 ns increments. All trajectory snapshots were aligned to the 0 ns starting structure, which is shown in gray for reference. The aligned snapshots are shown from (b) the side and (c) down the length of the oligomer.

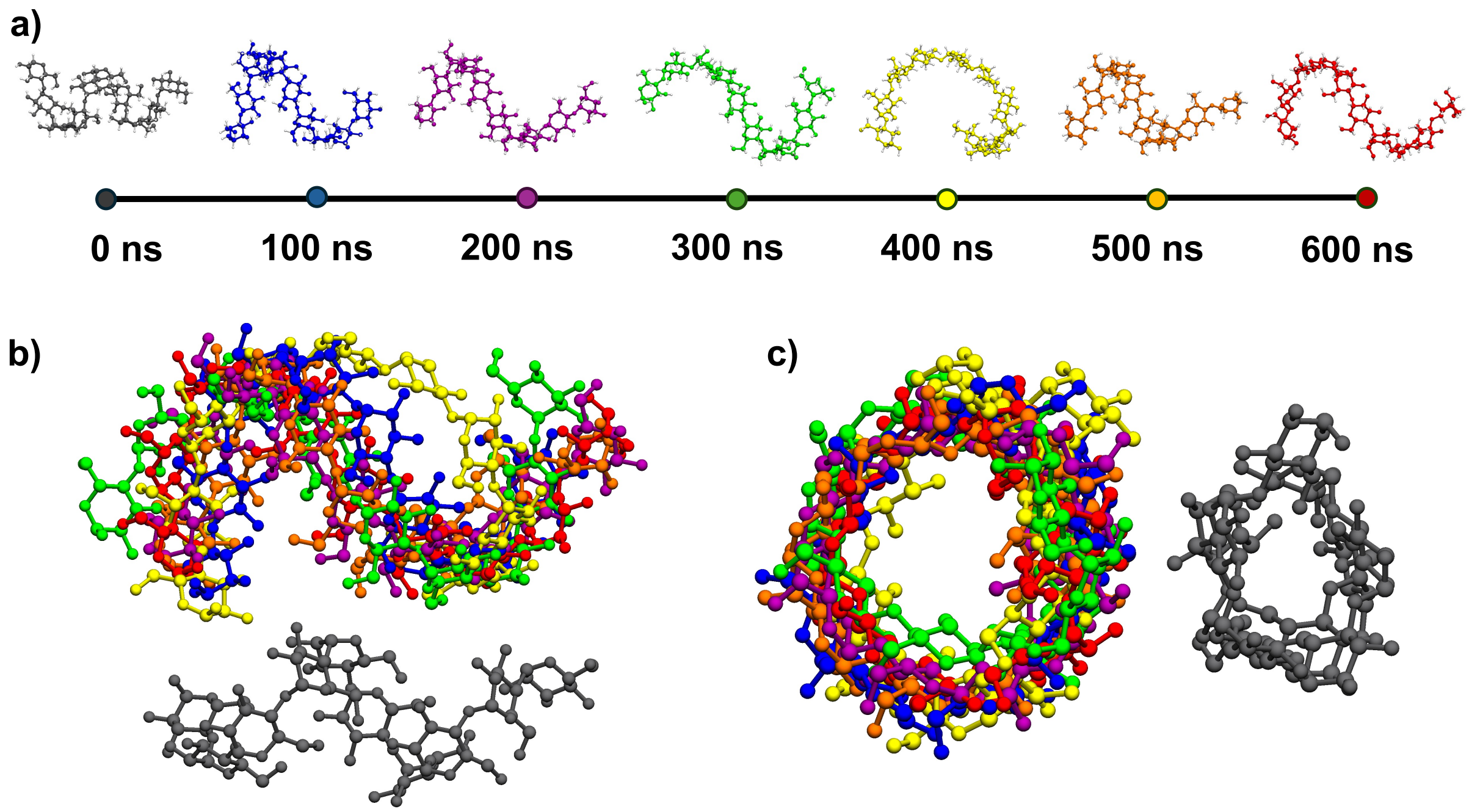

Figure S23: Representative trajectory snapshots of the 1.0-scaled α-D-glucose 10-unit oligomer. (a) Snapshot structures shown at 100 ns increments. All trajectory snapshots were aligned to the 0 ns starting structure, which is shown in gray for reference. The aligned snapshots are shown from (b) the side and (c) down the length of the oligomer.

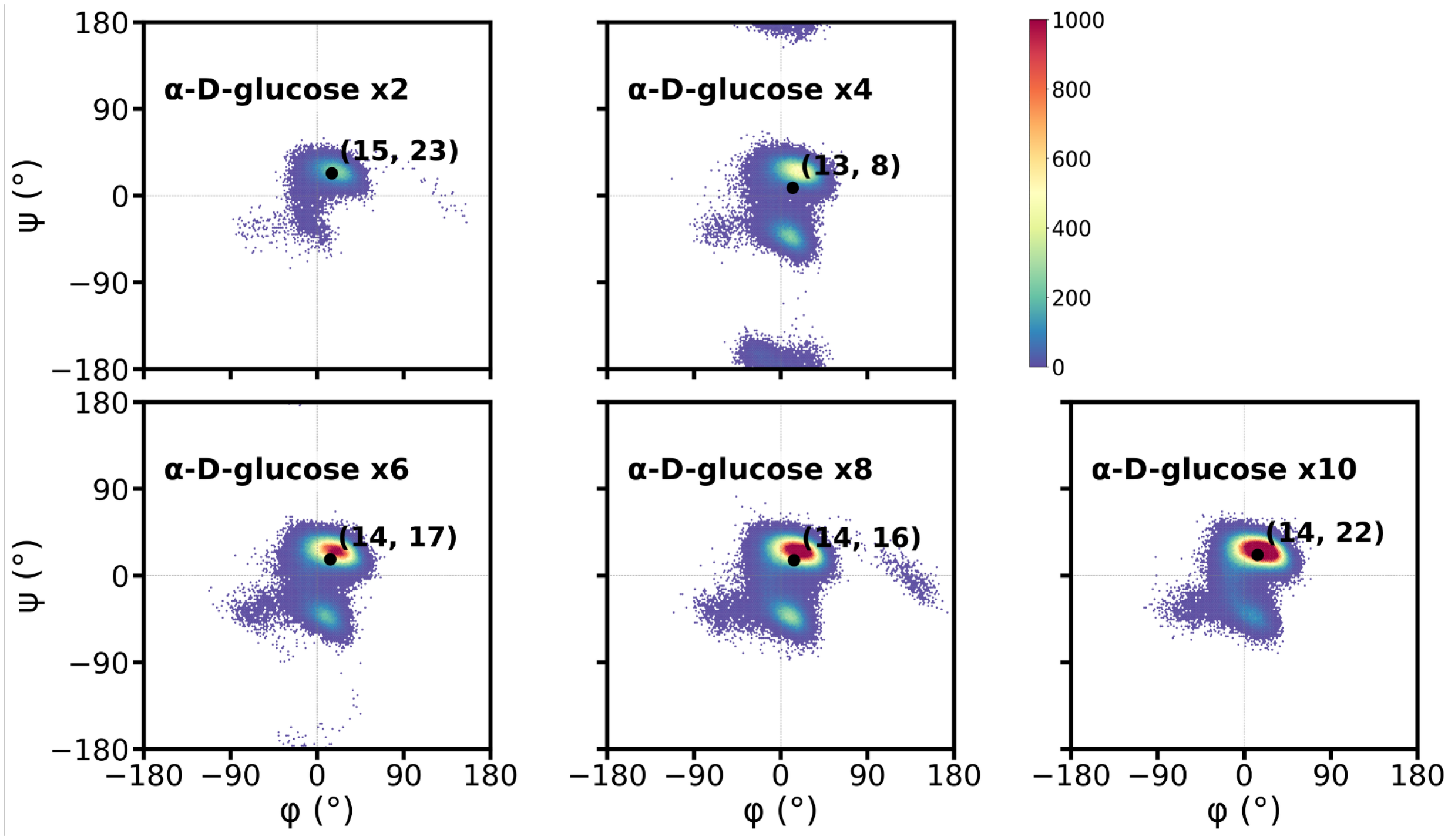

Figure S24: Density plots of the glycosidic dihedral angles ϕ_H (H1–C1–O–C4) and ψ_H (C1–O–C4–H4) for α-D-glucose oligomers with 0.7 oxygen quadrupole scaling. Panels show results for oligomers containing 2, 4, 6, 8, and 10 D-glucose units. The black point denotes the average ϕ_H/ψ_H angle pair for each distribution.

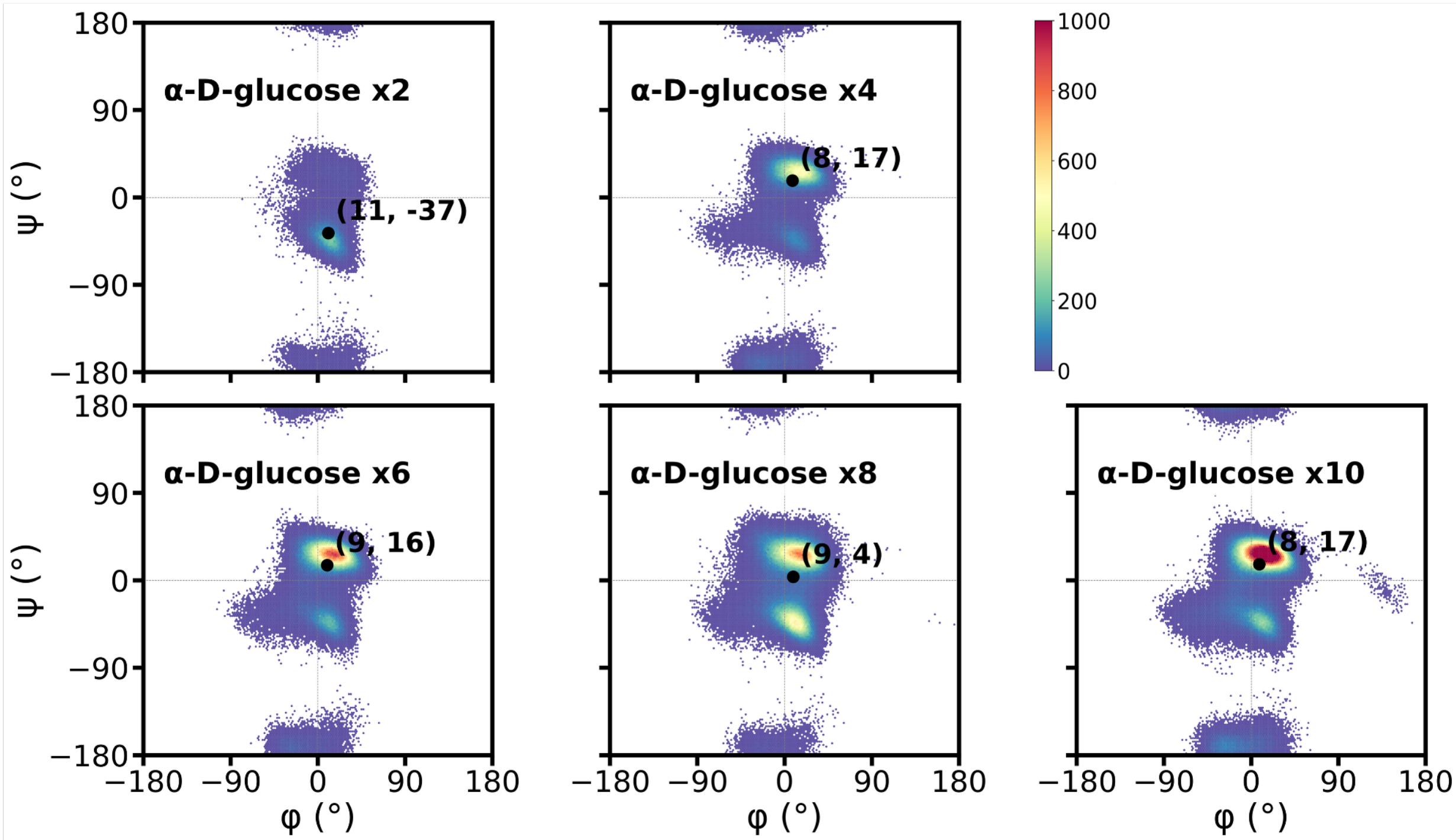

Figure S25: Density plots of the glycosidic dihedral angles ϕ_{H} (H1–C1–O–C4) and ψ_{H} (C1–O–C4–H4) for α-D-glucose oligomers with 1.0 oxygen quadrupole scaling. Panels show results for oligomers containing 2, 4, 6, 8, and 10 D-glucose units. The black point denotes the average $\phi_{\mathrm{H}}/\psi_{\mathrm{H}}$ angle pair for each distribution.

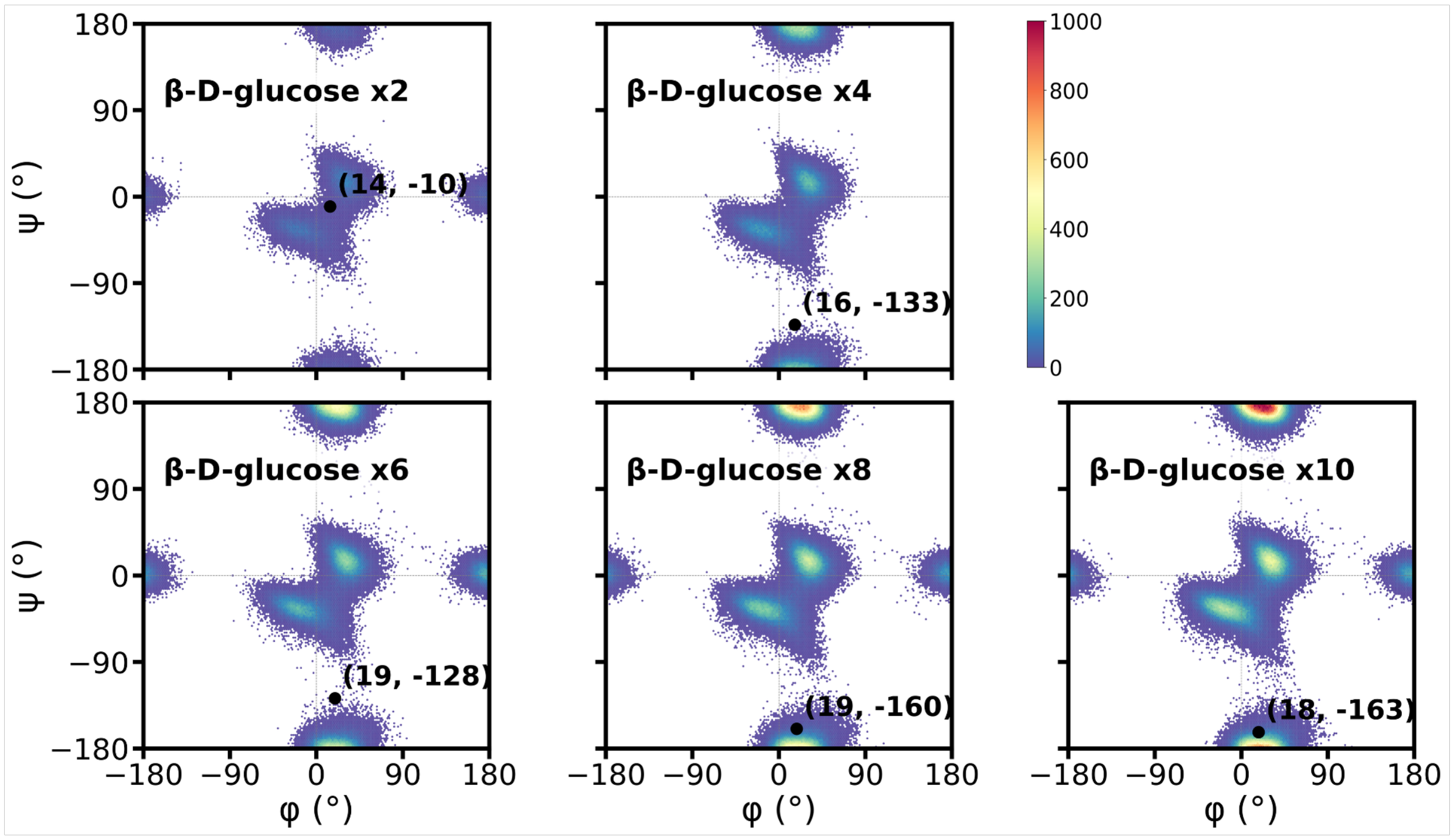

Figure S26: Density plots of the glycosidic dihedral angles ϕ_H (H1–C1–O–C4) and ψ_H (C1–O–C4–H4) for β-D-glucose oligomers with 0.7 oxygen quadrupole scaling. Panels show results for oligomers containing 2, 4, 6, 8, and 10 D-glucose units. The black point denotes the average ϕ_H/ψ_H angle pair for each distribution.

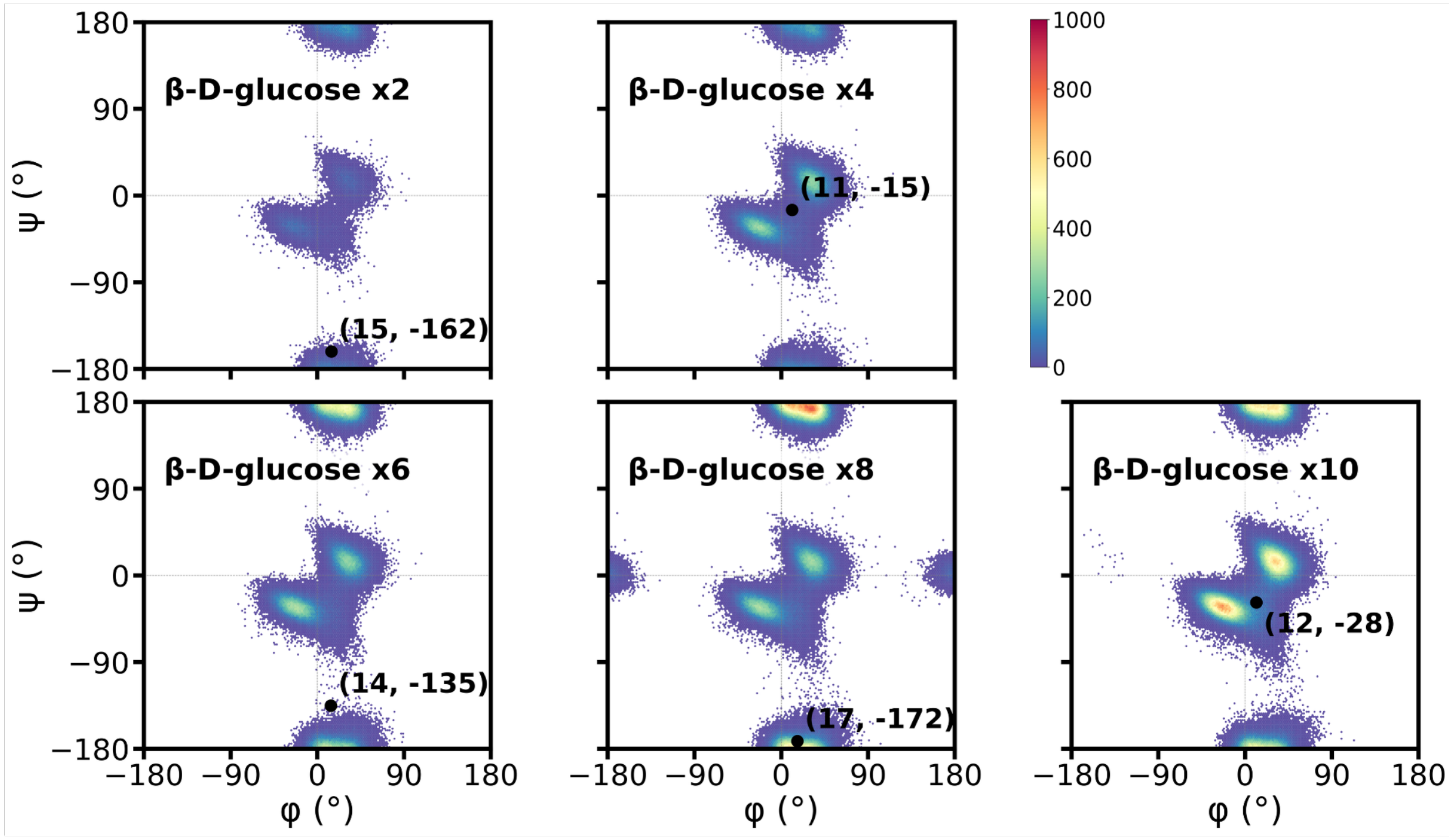

Figure S27: Density plots of the glycosidic dihedral angles ϕ_H (H1–C1–O–C4) and ψ_H (C1–O–C4–H4) for β-D-glucose oligomers with 1.0 oxygen quadrupole scaling. Panels show results for oligomers containing 2, 4, 6, 8, and 10 D-glucose units. The black point denotes the average ϕ_H/ψ_H angle pair for each distribution.

Table S3: Glycosidic three-bond NMR J-coupling constants for the α- and β-linked D-glucose dimers. The AMOEBA values were calculated from the molecular dynamics torsion-angle distributions using the Karplus equation from Tvaroška et al.[?] Experimental values are from the Cheetham et al. NMR study.[?] Experimental results are reported as the mean ± the reported experimental error, whereas AMOEBA results are reported as the mean ± the block standard error of the mean (SEM). All values are in Hz.

Anomer	Coupling	Experiment	AMOEBA 0.7	AMOEBA 1.0
α-D-glucose dimer	$^3J(\mathrm{H1, C4})$ from ϕ	4.12 ± 0.20	5.139 ± 0.016	4.938 ± 0.006
	$^3J(\mathrm{C1, H4})$ from ψ	4.68 ± 0.10	3.699 ± 0.145	4.600 ± 0.009
β-D-glucose dimer	$^3J(\mathrm{H1, C4})$ from ϕ	4.08 ± 0.04	4.725 ± 0.144	4.533 ± 0.029
	$^3J(\mathrm{C1, H4})$ from ψ	4.75 ± 0.25	4.880 ± 0.193	5.630 ± 0.336

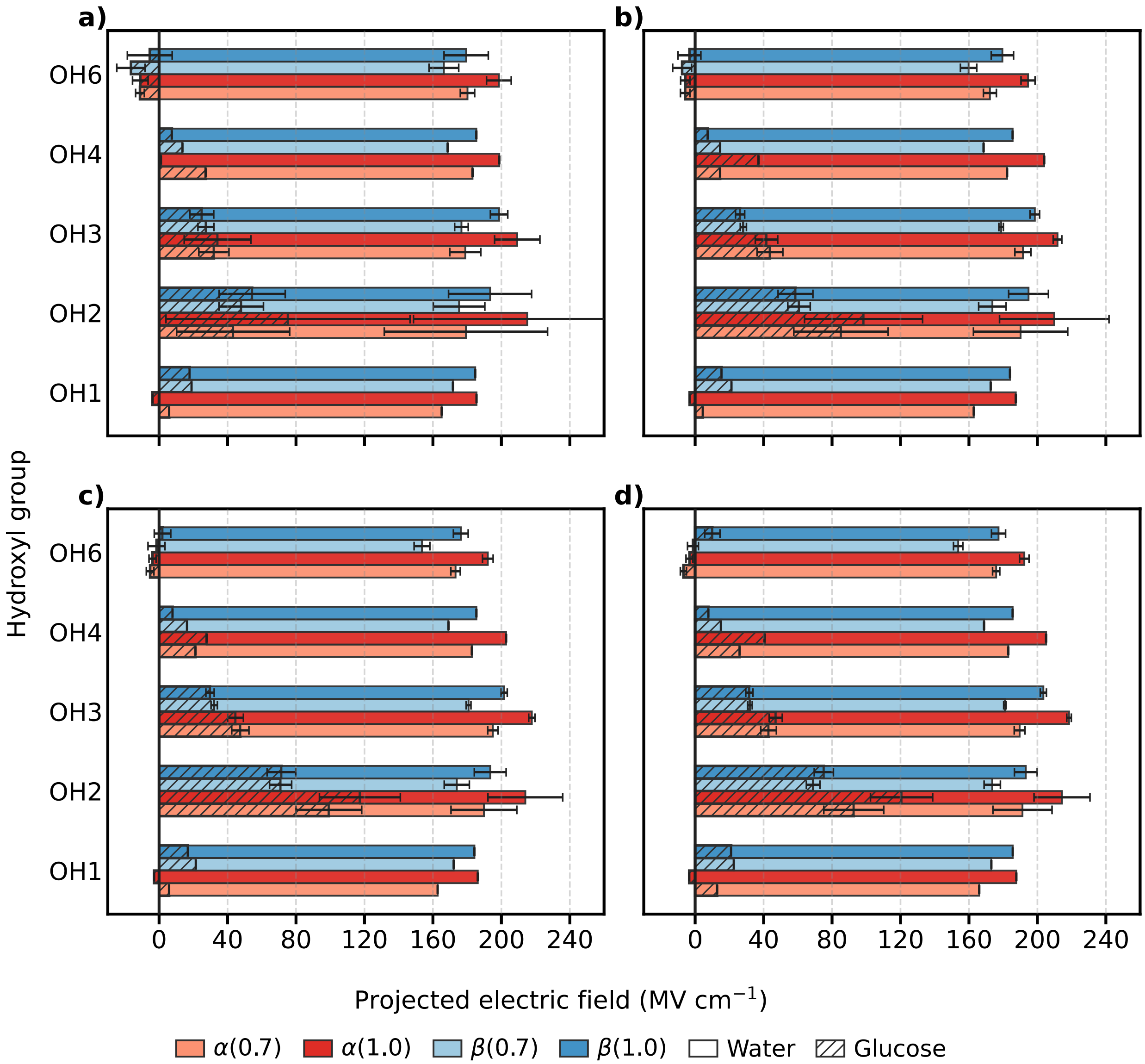

Figure S28: Decomposition of the projected electric field along each hydroxyl bond into water and intramolecular oligomer contributions for paired α- and β-D-glucose oligomers containing a) 2, b) 4, c) 6, and d) 8 D-glucose units. Solid bars show the water contribution, and hatched bars show the intramolecular oligomer contribution. Error bars represent the standard error across equivalent hydroxyl groups for repeated sites OH2, OH3, and OH6, and the standard error over trajectory frames for the non-repeated terminal sites OH1 and OH4.

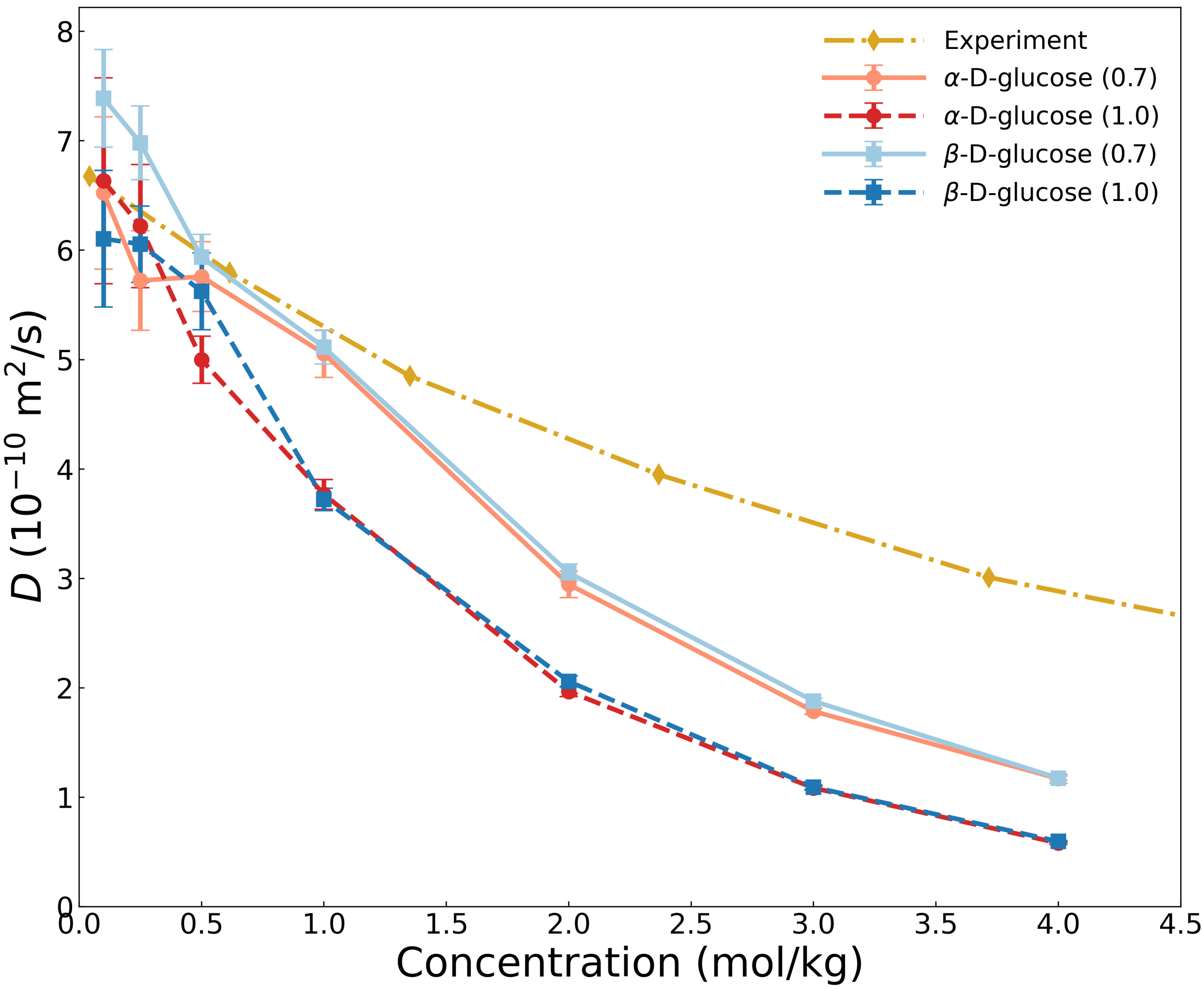

Figure S29: Finite-size-corrected diffusion coefficients of D-glucose as a function of D-glucose concentration, with results separated by α- and β-anomers.

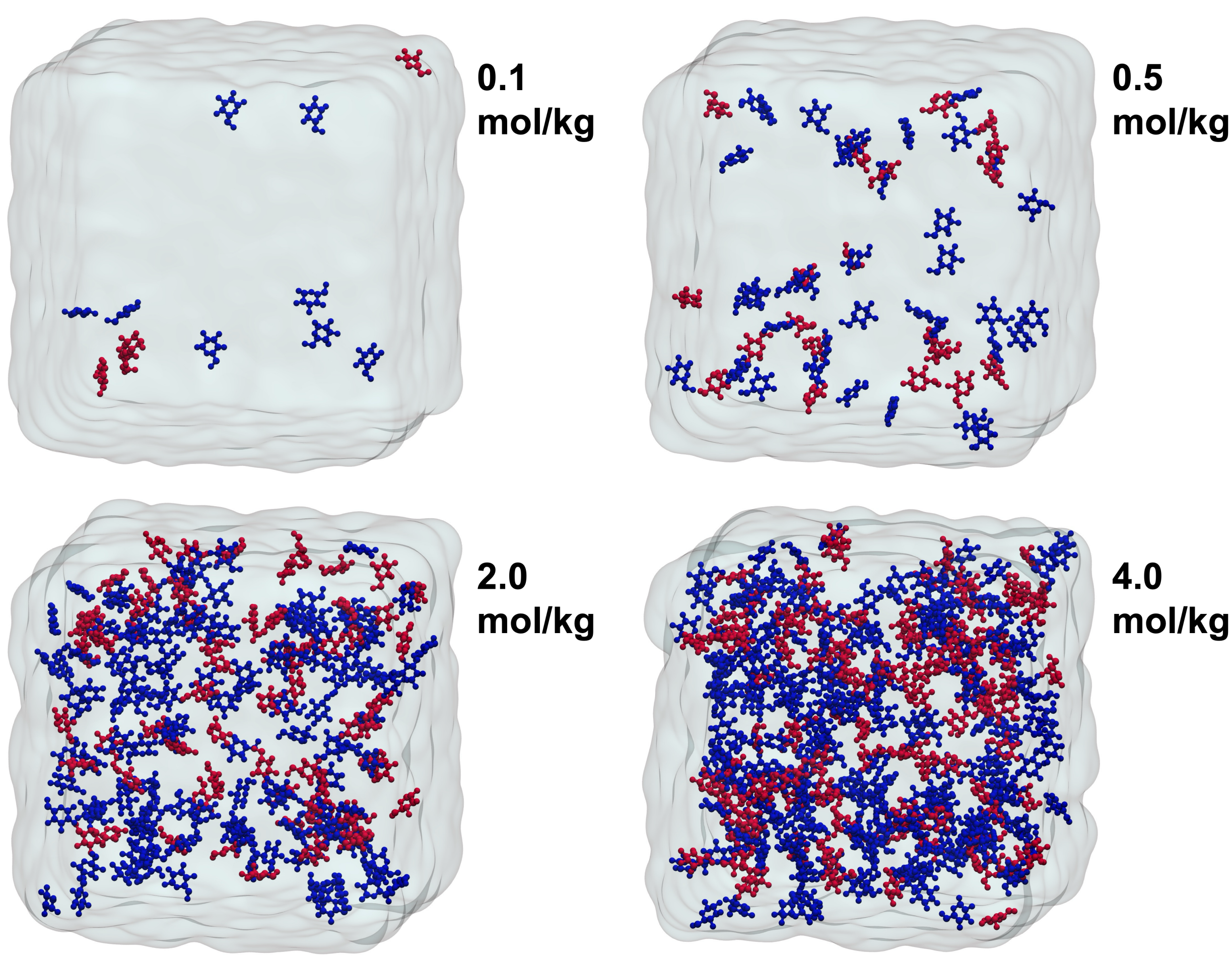

Figure S30: Representative snapshots of D-glucose concentration systems at 0.1, 0.5, 2.0, and 4.0 mol/kg

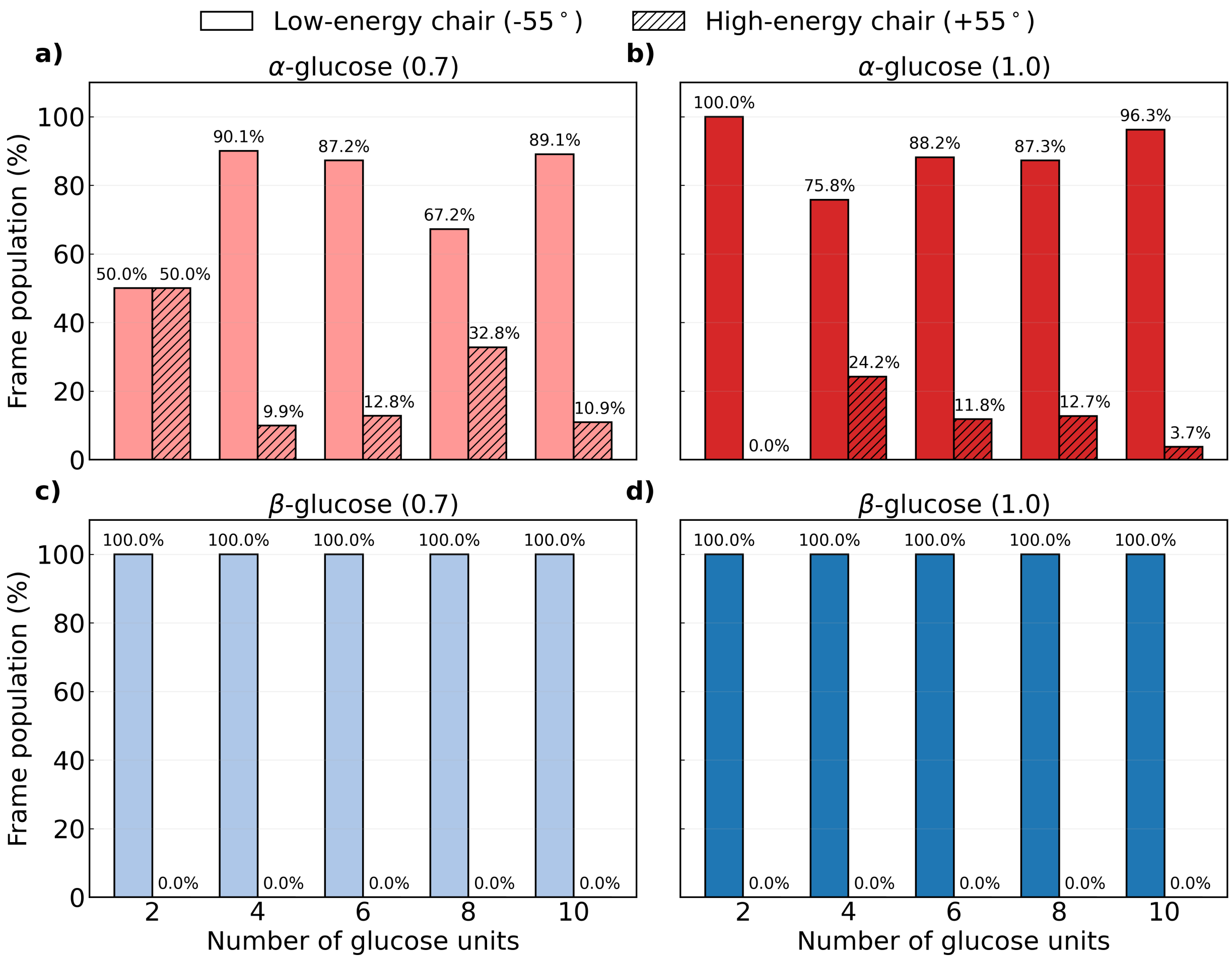

Figure S31: Populations of low- and high-energy chair conformations for α- and β-D-glucose oligomers at oxygen quadrupole scaling factors of 0.7 and 1.0. Panels show (a) α, 0.7; (b) α, 1.0; (c) β, 0.7; and (d) β, 1.0 for oligomers containing 2–10 D-glucose units. Unhatched and hatched bars denote the low-energy ($-55°$) and high-energy ($+55°$) chair populations, respectively. Populations were calculated by assigning each C1–C2–C3–C4 torsion to the nearest chair reference using circular angular distance and pooling assignments across all glucose units within each oligomer length.
